# Decoupling Intrinsic Metal Ion Reduction Rates from Structural Outcomes in Multimetallic Nanoparticles

**DOI:** 10.1021/jacs.4c13826

**Published:** 2024-12-09

**Authors:** Jacob H. Smith, Qi Luo, Shelby L. Millheim, Jill E. Millstone

**Affiliations:** † Department of Chemistry, University of Pittsburgh, 219 Parkman Avenue, Pittsburgh, Pennsylvania 15260, United States; ‡ Department of Chemical and Petroleum Engineering, University of Pittsburgh, Pittsburgh, Pennsylvania 15260, United States; § Department of Mechanical Engineering and Materials Science, University of Pittsburgh, Pittsburgh, Pennsylvania 15260, United States

## Abstract

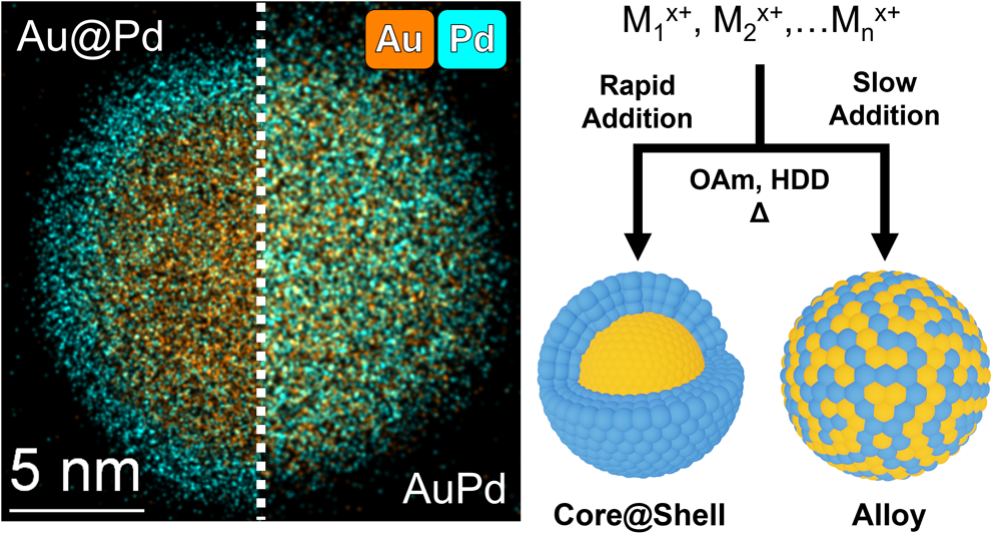

Simultaneously controlling both stoichiometry and atom arrangement during the synthesis of multimetallic nanoparticles is often challenging, especially when the desired metal precursors exhibit large differences in their intrinsic reduction kinetics. In such cases, traditional synthetic methods often lead to the formation of exclusively phase-segregated structures. In this study, we demonstrate that the relative reduction kinetics of the metal precursors can be manipulated independently of their intrinsic differences in reduction rates by modulating the instantaneous concentrations of the metal cation precursors. We achieve this control by adjusting the precursor addition rate, which decouples chemical ordering outcomes from differences in precursor reduction kinetics. To guide these experiments, we describe a quantitative model to determine how metal ion reduction rates evolve with variations in the precursor addition rate and thereby predict optimal conditions for the synthesis of multimetallic nanoparticles with precise structural and compositional outcomes. We demonstrate the efficacy of this model experimentally by synthesizing both core@shell and alloyed nanoparticles with stoichiometric control using the same metal ion precursors in two different bimetallic systems (Au–Pd and Au–Pt) as well as in a quinary metal system (Co, Ni, Cu, Pd, and Pt). This approach enables the design of nanoparticle architectures independent of intrinsic differences in metal ion reduction potentials of the constituent metals while maintaining both stoichiometric and structural control.

## Introduction

For nanoparticles, parameters such as size and shape work in concert with chemical structure to create materials with unique chemical and physical properties.^1−5^ To control chemical ordering at this length scale, a wide variety of methods have been employed depending on the targeted structures and their intended uses ranging from metal-oxide support-based synthesis^6−10^ to laser ablation methodologies.^11−15^ The degree to which each synthetic technique yields systematic control over multimetallic nanoparticle (NP) stoichiometry and atom arrangement varies widely. However, unifying themes have emerged. For example, relative metal ion reduction rates have been shown to predict particle outcomes in multimetallic NP syntheses when using both one-pot and seed mediated strategies.^16−30^ Specifically, when two metal ion precursors exhibit similar reduction rates, alloyed NPs can be synthesized via one-pot, co-reduction strategies.^16,19,20,31,32^ On the other hand, core@shell structures are typically observed in these syntheses when the two metal ion precursors exhibit large differences in reduction rates.^22,24,26,33,34^ Core@shell structures can also be obtained via seed-meditated techniques, where a core is created first and then a shell is grown onto that core either using the same or different reaction conditions.^16,35−37^ Further, in cases where the initial reduction rates of metal ion precursors are not sufficiently similar, deviations from nominal stoichiometry are observed.^16,21,38−41^

To address these challenges, strategies have been developed to engineer metal ion reduction rates via modifications to either the metal precursor (e.g., the selection of alternative metal salts^22,42−46^ or the addition of various complexing agents^26,47−50^) or to the reducing agent chemistry.^31,51−54^ These approaches have been successful in producing a variety of bimetallic NP compositions, stoichiometries, and chemical orderings. However, this strategy is limited by the availability of suitable precursors for any given pair of metals and becomes more difficult to sustain as the number of metals to be incorporated increases. An alternative approach to optimize these reduction rates is to adjust the relative concentration of metal precursors, directly impacting the initial reduction rates as described by classic rate law relationships.^21,41,55−59^ Although adjusting precursor concentration in this way can balance kinetic differences in some systems, this strategy can limit stochiometric control.

Here, we investigate an approach that introduces metal ion precursors to a reaction mixture at varying rates, such that controlling these rates can be used to effectively decouple intrinsic differences in reduction kinetics from the final NP outcomes. Specifically, we first develop an experimental and theoretical approach using controlled precursor addition rates to modulate the instantaneous concentration of metal precursors, and thereby their instantaneous reduction rates. We then use this approach to successfully synthesize both core@shell and alloy NPs of a given bimetallic composition with stoichiometric control of both elements within either architecture. This approach is demonstrated using metal combinations that exhibit both favorable (Au–Pd)^60^ and unfavorable (Au–Pt)^61^ mixing behavior in the bulk, as well as for a multi principle element system using 5 metals (Co, Ni, Cu, Pd, and Pt). By decoupling the intrinsic differences in precursor reduction kinetics from the final NP architecture, our results demonstrate a potentially powerful new approach to predicting and achieving increasingly more complex multimetallic NPs while maintaining both stoichiometric and structural control.

## Results and Discussion

Our overarching goal is to modulate the instantaneous concentrations of metal cation precursors such that the stoichiometry and metal ordering in the resulting NPs can be controlled independently from the reduction potentials of the constituent metals. To develop our approach, we first investigated bimetallic systems of Au–Pd and Au–Pt and used a hot injection synthesis with oleylamine as the solvent, capping ligand, and co-reductant along with 1,2-hexadecanediol. These compositions and conditions were selected because the speciation and redox behavior of the metal chloride salts in oleylamine are well documented,^62−65^ and they are expected to exhibit large differences in reduction potential, which is ideal for our study. The comparison of Au–Pd and Au–Pt outcomes also allows us to test the role of bulk metal miscibility, where Au and Pd are broadly soluble but Au and Pt exhibit only sparing solubility according to their phase diagrams and consistent with their enthalpies of mixing.^60,61^ We also quantified the relative reduction rates of these precursors under our specific synthetic conditions using a combination of ICP-OES and UV–vis spectroscopy, where the relative reduction rates for Au:Pd and Au:Pt were measured to be 5.26 and 20.09, respectively (Figures S1–S3, Table S1).

Using these reduction rates, we could then calculate the evolution of instantaneous concentrations and instantaneous reduction rates for the metal precursors as a function of the precursor addition rate as follows. Previous investigations on colloidal metal NP syntheses via solution reduction indicate that these reactions follow a second-order rate law, dependent on the concentrations of the metal precursor ([*A*^*n*+^]) and the reducing agent ([Red]).^27,28,47^ When reducing agent is used in large excess (i.e., [Red] ≫ [*A*^*n*+^]), such that [Red] remains approximately constant throughout the duration of the reaction, the reaction rate can be approximated as a pseudo-first-order reaction described as:
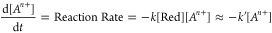
1where *k’* is the associated rate constant of the reaction and is dependent on the specific synthetic parameters (e.g., the identity of the metal precursor, the specific reducing agent(s), the solvent identity, and the reaction temperature).^28^ This approximation is valid even when multiple reducing agents are present, provided they are all in large excess.^24^ The instantaneous concentration of unreacted precursor at any time (*t*) during the reaction can be obtained by integrating eq 1 with respect to time yielding eq 2:
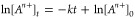
2

If the metal precursor is instead titrated into the reaction solution, the rate of change in the metal precursor concentration, , becomes a combination of the rate at which the metal precursor is added to the reaction mixture and the rate at which the precursor is consumed in the reaction. Then, the rate of change in the metal precursor concentration can be expressed quantitively as the sum of the precursor addition rate and the precursor reaction rate yielding eq 3. Equation 3 is represented as a piece wise function to describe two distinct periods: one corresponding to the metal addition period and the other corresponding to the period after metal addition is complete.
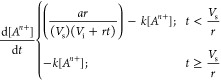
3Here, *a* is the total amount of each precursor added (mol), *V*_s_ (mL) is the volume of the precursor solution injected, *r* (mL/h) is the injection rate, and *V*_i_ (mL) is the initial volume in the reaction flask. The total volume at any time (*t*) is the sum of the initial volume (*V*_i_) and the volume added over time (*r* × *t*). Once , the metal addition period is over and the rate of addition becomes zero, such that the rate of change in each metal precursor concentration is described solely by its reaction rate as described in eq 1. The instantaneous concentration of either metal precursor at any time during the reaction can be obtained by integrating eq 3 with respect to time.

**Figure 1:**
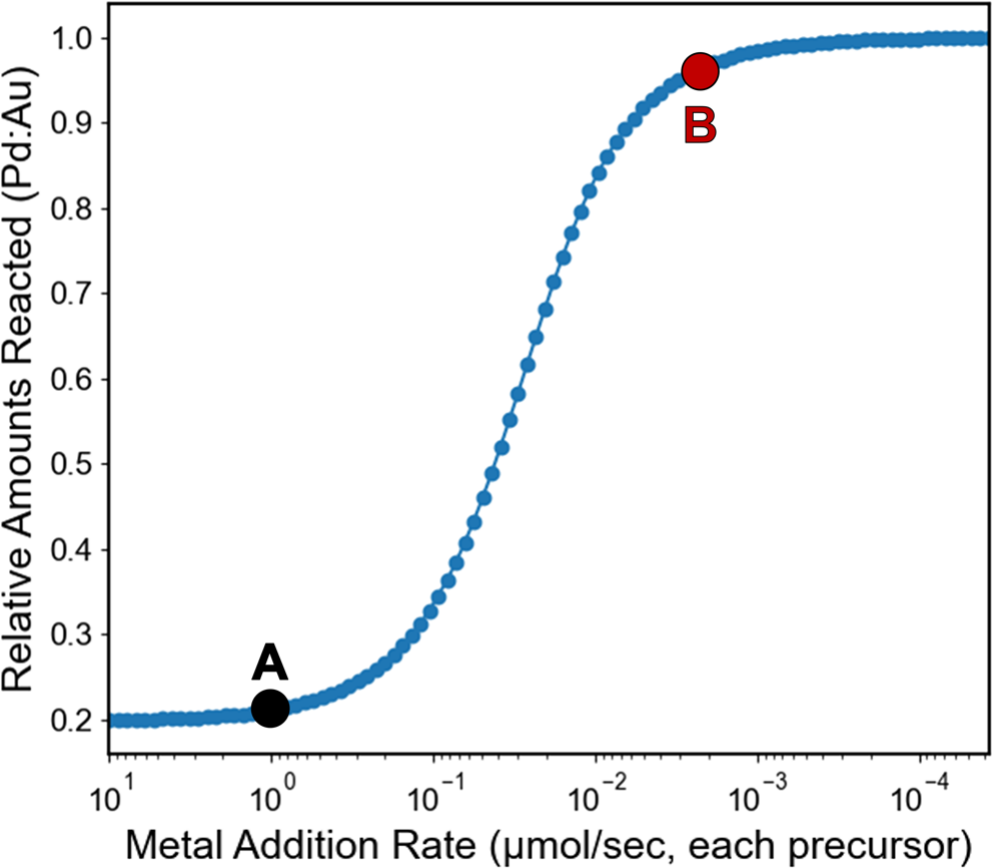
Plot of the predicted relative amounts of Au^3+^ and Pd^2+^ reacted during a given metal addition period as a function of precursor addition rate. Each point represents a simulation corresponding to a different addition rate for a given metal ion precursor. The black circle (Point A) corresponds to a rapid addition rate of 1000 nmol/s (Pd:Au = 0.22) and the red circle (Point B) corresponds to a slow addition rate of 0.2 nmol/s (Pd:Au = 0.95).

Using this framework and the determined reduction rate constants (Table S1), we were then able to simulate how the instantaneous concentrations and corresponding reduction rates of the metal precursors should evolve as a function of precursor addition rate into a given reaction solution, assuming a constant total amount of metal precursor for all addition rates (Figures S4, S5). For example, we calculated the relative amounts of Au^3+^ and Pd^2+^ that have reacted during a given metal addition period by integrating eq 3 from *t* = 0 to . In this example, we considered 100 different addition rates spanning 6 orders of magnitude (Figure 1). Here, low ratios of reacted metals indicate that Pd^2+^ reduction lags behind Au^3+^ reduction, as there remains a significant concentration of unreacted Pd^2+^ in solution after metal addition ceases. Conversely, ratios of reacted metals closer to parity suggest that Au^3+^ and Pd^2+^ are reducing simultaneously throughout the metal addition period associated with that precursor addition rate. We hypothesized that when most NP formation occurs during the simultaneous reduction of Au^3+^ and Pd^2+^, the formation of alloyed NPs would be favored, and this condition is met when addition rates are slower (e.g., Point B in Figure 1). Further, it is important to note that this condition would not require that the reduction rates of the two metals be at parity (*vide infra*).

**Figure 2:**
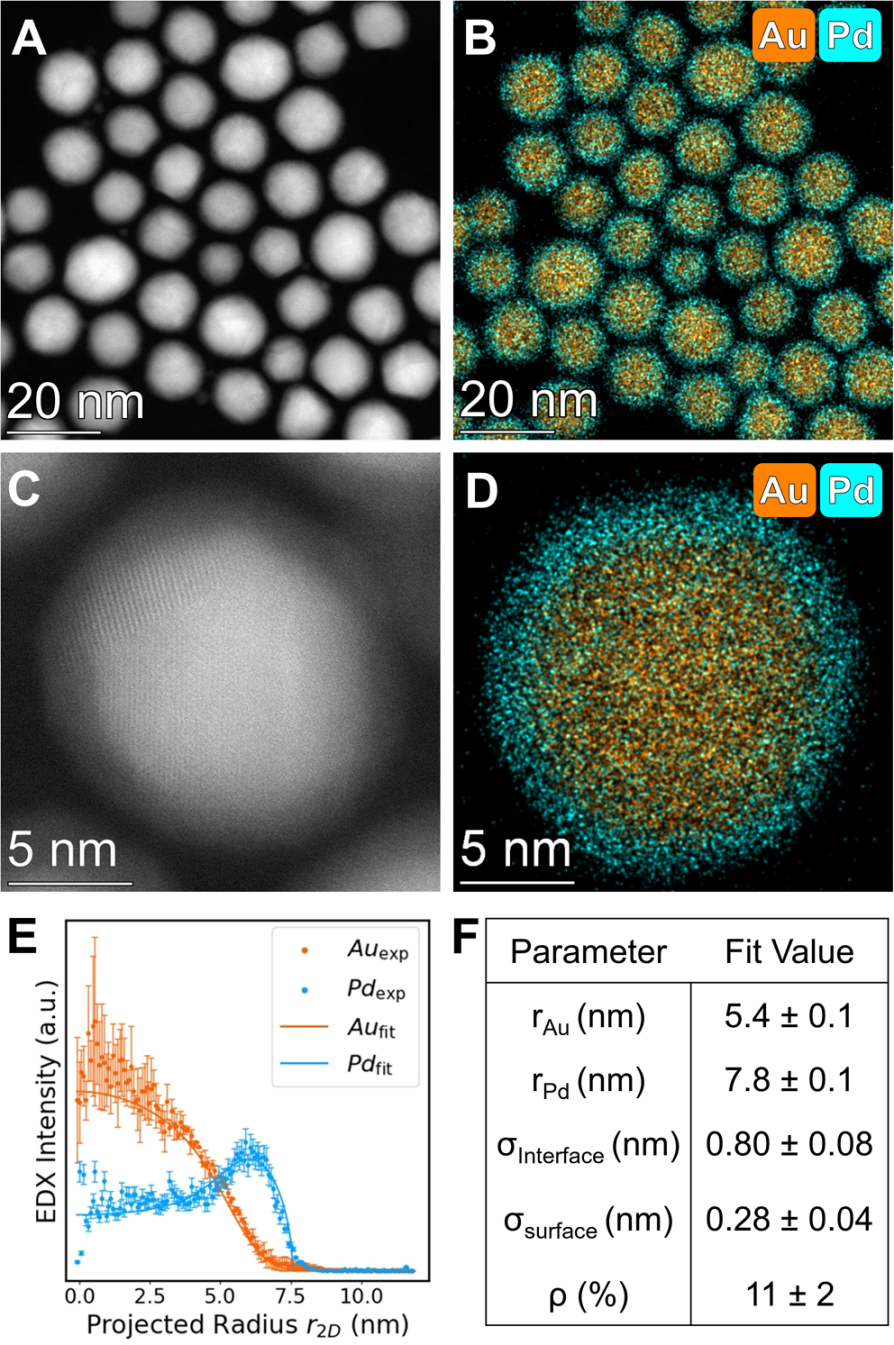
Structural characterization of Au@Pd NPs produced using the 1000 nmol/s addition rate. (A) Low magnification HAADF-STEM image and (B) corresponding EDX map of Au@Pd NPs. (C) High magnification HAADF-STEM image showing a twin structure and representative of crystal structures observed in these particles (Figure S8). (D) EDX map of a single Au@Pd NP. (E) Experimental radial profiles of each element within the NP (points) and corresponding analytical fit to the data (solid lines). (F) Optimized parameters derived from the fit. Palladium signals (Pd Kα) are displayed in blue and gold signals (Au Lα) are displayed in orange.

To test these predictions, we compared the nanoparticle outcomes from precursor addition rates that yield both high and low ratios of reacted metal precursor for Au–Pd and Au–Pt systems (Figure 1, points A and B, 1000 nmol/s and 0.2 nmol/s, respectively; Figure S6 points C and D, 1000 nmol/s and 0.2 nmol/s, respectively). In the case of the rapid addition (1000 nmol/s) of Au^3+^ and Pd^2+^, we observed core@shell structures uniform in size, shape, stoichiometry, and atom arrangement. Low magnification HAADF-STEM and EDX images (Figure 2A,B) show the Au@Pd core@shell NPs, averaging 12.4 ± 1.6 nm in diameter (Figure S7**)**. This outcome is consistent with sequential metal reduction commonly observed in co-reduction syntheses involving precursors with large differences in reduction potential as is the case here (Δ*E*_red_ for [AuCl_4_]^1–^/[Pd(OAm)_4_]^2+^ = +1.0 V).^63,64^ These results are also consistent with our experimentally determined reduction rate constants (Table S1), where the more quickly reducing metal, Au, forms the NP core followed by reduction of the more slowly reducing metal, Pd, as a Pd shell. Interestingly, we note that comparable monometallic Pd NPs were not observed in these reactions, likely due to the favorability of heterogeneous vs homogeneous nucleation and the broad availability of already formed Au NP surfaces. High magnification HAADF-STEM images (Figures 2C, S8) revealed a twinned crystal structure extending through both the core and shell, characteristic of NPs with an fcc crystal structure and indicative of epitaxial deposition of the Pd shell (Figure S8).^66,67^ Compositional analyses from both ensemble (ICP-OES, 51 ± 2% Au) and single particle (EDX, 54 ± 4% Au) measurements were in good agreement with the targeted 50:50 stoichiometry.

To analyze the spatial distribution of the elements within the NP architectures, we measured the element distributions using a radial profile analysis, and then used a literature technique^68^ to extract quantitative estimates of core and shell dimensions from the radial profiles. Briefly, by leveraging the pseudospherical geometry of these NPs, the 2D projections obtained from the EDX images were analyzed in cylindrical coordinates. These data were then fit to a model that allows one to extract three-dimensional structural parameters, in order to quantitatively estimate features such as the outer radii of the core and shell materials (*r*_core_, *r*_shell_), the interface diffusivity between the core and shell (σ_interface_), and the surface roughness (σ_surface_). As the NPs are synthesized via a co-reduction method, the ρ term is added to describe the amount of core material present in the shell (Figure 2E,F; for methodology details including error estimation in the model see SI eqs S1–S5, Figures S9, S10, and associated discussion). This analysis (Figure 2D–F) was performed on nine particles across three independent replicates, and revealed a sharp, well-defined interface, measured at 0.78 nm or roughly two-unit cells. Because this method cannot differentiate between interface diffusivity resulting from metal mixing (i.e., a region of alloyed AuPd and surface roughness at the core@shell interface) this thickness represents an upper limit. The distinct chemical interface observed using the rapid injection underscores the significant difference in reduction kinetics between Au^3+^ and Pd^2+^ precursors under these experimental conditions.

**Figure 3:**
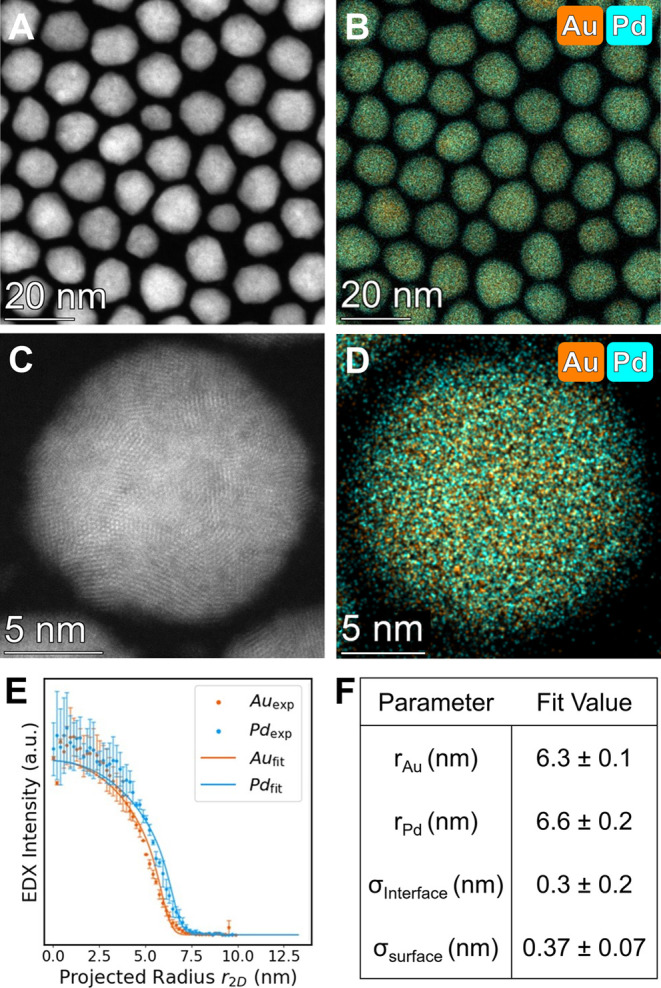
Structural characterization of AuPd alloy NPs produced from the 0.2 nmol/s addition rate. (A) Low magnification HAADF-STEM image and (B) corresponding EDX map of AuPd NPs. (C) High magnification HAADF-STEM image showing a polycrystalline NP representative of crystal structures observed in these particles (Figure S11). (D) EDX map of single AuPd alloy NPs. (E) Experimental radial profiles of each element within the NP (points) along with the analytical fit to the data (solid lines). (F) Optimized parameters derived from the fitting process. Palladium signals (Pd Kα) are displayed in blue and gold signals (Au Lα) are displayed in orange.

In contrast, when metal precursors were added at the slow addition rate (0.2 nmol/s), we obtained alloyed NPs uniform in size, shape, stoichiometry, and atom arrangement. Low magnification HAADF-STEM and EDX imaging (Figure 3A,B) revealed the formation of alloyed AuPd NPs, averaging 10.7 ± 1.7 nm in diameter (Figure S7). High magnification images (Figures 3C, S11) are also consistent with alloys as evidenced by the uniform contrast throughout the particle architecture. Compositional analyses from both ensemble (ICP-OES, 53 ± 2% Au) and single particle (EDX, 50 ± 2% Au) techniques agreed with the nominal 50:50 stoichiometry, indicating that metal stoichiometry too may be controllable using this approach (*vide infra*). Radial profile analysis of the alloyed NPs (Figure 3D–F) confirmed a uniform distribution of both elements throughout the particle. The difference in chemical ordering between the two addition rates was further indicated at the ensemble level using UV–vis extinction spectroscopy (Figure S12) and PXRD analysis (Figures S13–S15, Tables S3 and S4). The observation of alloyed NPs when using slow addition rates, but otherwise identical experimental conditions to those used to produce core@shell structures above, supports our hypothesis that controlling the precursor addition rate can effectively overcome the differences between reduction kinetics of metal precursors and facilitate control of metal ordering in multimetallic NPs independent from their standard reduction potentials.

To determine whether the differences in chemical arrangements between the two precursor addition rate experiments could be attributed to variations in the reaction duration and subsequent exposure to elevated temperatures, we conducted a series of control experiments. First, we completed both syntheses at the standard temperature of 140 °C, and then raised their temperatures to 220 °Cfor an additional 6 h. In the case of core@shell NPs, the core@shell structure was preserved in all cases, and no evidence of alloying was observed (Figure S16). Likewise, no evidence of metal segregation was observed in the alloyed NP case. Taken together, these results support that the observed differences in chemical arrangements do not arise from heat-induced changes due to reaction times. This conclusion may also be expected since the temperature range typically required for solid-state diffusion over this length scale (both in the bulk and in NPs) is much greater than the temperatures used in our experiments bulk and NP.^69−71^

We next compared the impacts of rapid and slow metal precursor addition in an analogous Au–Pt system using the same synthetic approach. We chose the Au–Pt combination in order to test whether our method could control chemical ordering even for a metal mixture that is known to be largely immiscible in the bulk (i.e., one having a positive enthalpy of mixing),^61,69,72^ and thereby also probing a potential role of bulk metal miscibility in the formation of these, and possibly other, bimetallic NPs. Briefly, using the same model to predict optimum precursor addition rates (Figure S6, and *vide supra*), we compared NP outcomes using both rapid (1000 nmol/s) and slow (0.2 nmol/s) metal ion addition rates. Consistent with the measured reduction rate constants and the results obtained with the Au–Pd combination, rapid precursor addition resulted in the formation of Au@Pt structures whereas slow precursor addition led to the formation of alloyed AuPt NPs. Further, both morphological outcomes were in good agreement with the targeted 50:50 stoichiometry. These results were confirmed by the same analyses described above including HAADF-STEM, EDX, and ICP-OES (Figures S17, S18). The observation of both core@shell and alloyed morphologies, as well as their close match between nominal and resulting metal stoichiometries, suggest that bulk metal miscibility may either not be a predictor of metal mixing in these NPs or that bulk miscibility challenges can be mitigated using this synthetic approach.

Taken together, the above results support our hypothesis regarding the importance of simultaneous metal ion precursor reduction over the duration of NP formation as well as our model for predicting optimal precursor addition rates for a given chemical ordering. Specifically, we demonstrated that using a slow precursor addition rate could produce alloyed NPs even in cases where intrinsic differences in reduction kinetics between the precursors (and their standard reduction potentials) indicate that only core@shell morphologies should be accessible. These results suggest a subtle but important distinction to hypotheses concerning the formation of bimetallic NPs. Conventionally, achieving desired metal mixing outcomes is thought to require optimizing the reduction rates of metal precursors (often approximated by differences in Δ*E*_red_ between the constituent metals). For one-pot syntheses, this optimization involves selecting precursors with similar reduction kinetics to obtain alloyed structures or using precursors with sufficiently different reduction kinetics to obtain core@shell NPs. However, our results suggest that alloy formation does not necessarily require the reduction kinetics of the metal ions to match, but rather requires the synchronization of their reduction during NP formation. To test this distinction, we synthesized Au–Pd and Au–Pt at different stoichiometries using both slow and fast metal ion addition rates. If relative metal ion reduction rate is the primary driver of chemical ordering in the final NPs, then changing the nominal stoichiometries of the metals added should change the observed chemical orderings from what we saw empirically at the 50:50 molar ratios above (i.e., the concentrations of the two metals are now different, and therefore their relative reduction rates are now different by definition, eq 1, Figure S19). On the other hand, if the observed chemical orderings are preserved at different stoichiometries, then the relative metal ion reduction rates alone cannot explain the chemical ordering outcomes, which we explain is better predicted by identifying when metal ion reduction is synchronized.

**Figure 4:**
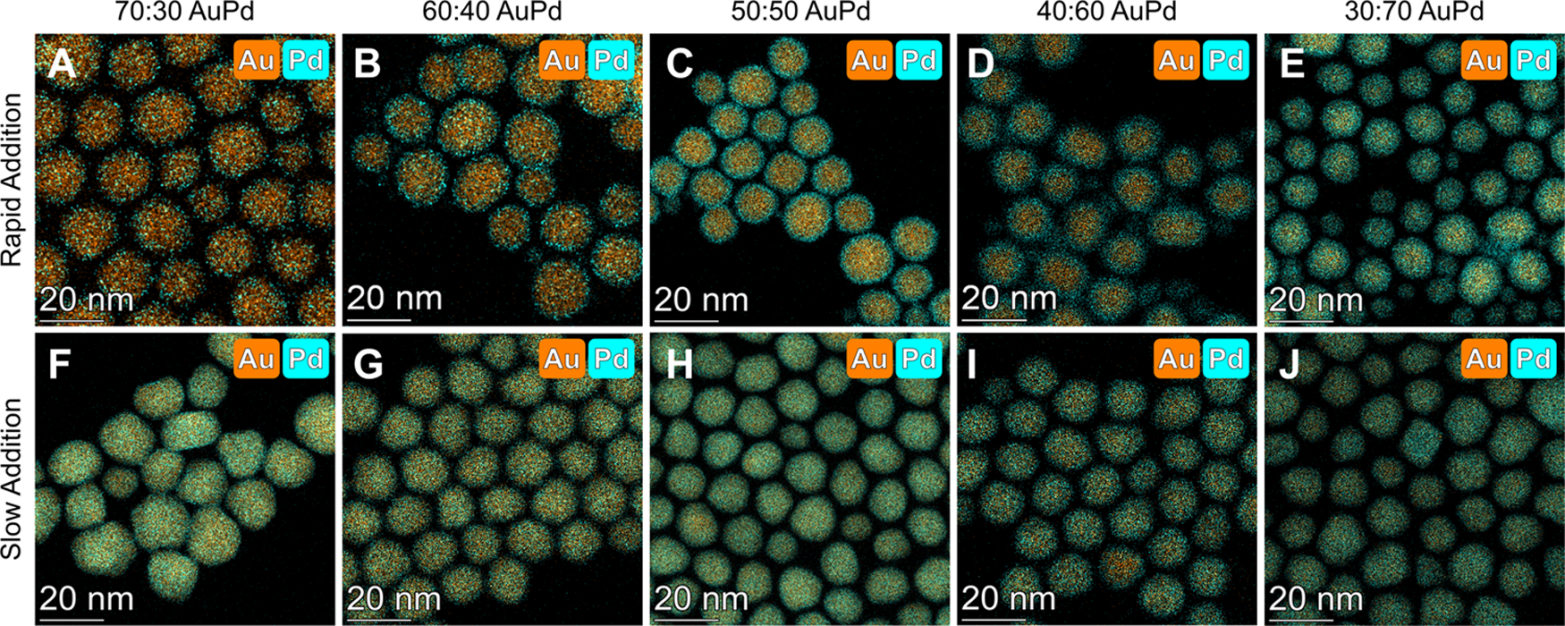
EDX images of AuPd NPs produced using different molar feed ratios of Au:Pd and either (A–E) a rapid addition rate (2000 nmol/s, total precursor) or (F–J) a slow addition rate (0.4 nmol/s, total precursor). Palladium signals (Pd Kα) are displayed in blue and gold signals (Au Lα) are displayed in orange.

Different stoichiometries were targeted by varying the molar ratio of Au:Pd or Au:Pt from 30 to 70% Au, while maintaining the same total metal concentration and reaction volume used in the 50:50 case described above. For all molar ratios of Au:Pd and Au:Pt tested, rapid addition of the metal precursors (2,000 nmol/s, total precursor) produced core@shell NPs (Figures 4A–E, S20A–C). Changing the ratio of Au to the other metal primarily impacted the shell thickness, with more secondary metal resulting in thicker shells of that metal. In contrast, when the precursors were added slowly (0.4 nmol/s, total precursor), all ratios resulted in the formation of alloyed NPs (Figures 4F–J, S20D–F).

**Figure 5:**
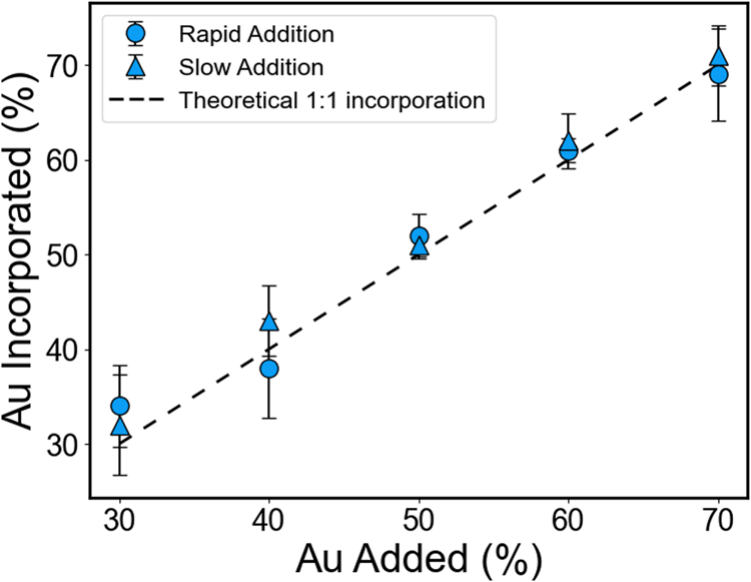
Percent Au incorporated into AuPd NP samples as a function of initial molar percent Au added to the synthesis for both the rapid (circles) and slow (triangles) addition rates. Results show good agreement between % added and % incorporated for both slow and fast addition rates. The dashed line represents the theoretical stoichiometry assuming 1:1 incorporation.

The compositional outcomes of the resulting NPs were then characterized using a combination of ensemble (ICP-OES) and single particle (EDX) analyses. In both analyses, NP stoichiometries matched within error to the targeted stoichiometries for all samples, regardless of addition rate (Figures 5, S21, Tables S5, S6). These results suggest that techniques to modulate simultaneous reduction, rather than techniques to achieve parity between metal ion reduction rates, can be used to simultaneously control both stoichiometry and chemical ordering in the final NPs, and remove the need to develop alternative metal ion precursors or reducing agent chemistries in those cases.

Encouraged by the agreement between model and experiment above, a clear next question is the extent to which this method could enhance structural control in more complex systems. To assess the efficacy of our approach beyond the synthesis of bimetallic NPs, we aimed to synthesize a quinary (i.e., high entropy) system, CoNiCuPdPt. This system was selected because it incorporates 2 noble and 3 non-noble metals, introducing a wide range of standard reduction potentials^63,73^ and atomic radii^73^ (Table S7) and, to our knowledge, no alloyed phases of this combination have been previously reported at the nanoscale.

**Figure 6:**
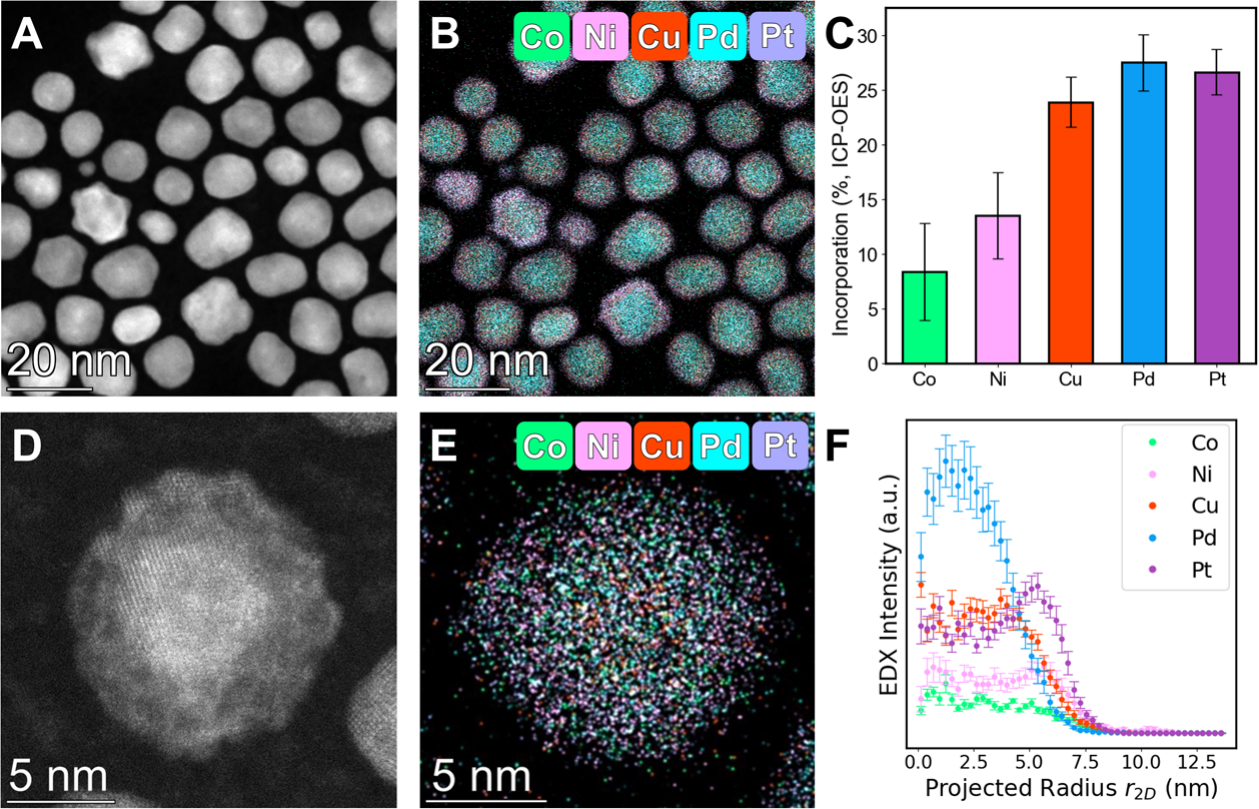
Structural characterization of 5 component CoNiCuPdPt NPs produced using the rapid addition rate of 10,000 nmol/s. (A) Low magnification HAADF-STEM image and (B) corresponding EDX map of CoNiCuPdPt NPs. (C) Bar graphs of the incorporation of each metal as measured by ICP-OES (D) High magnification HAADF-STEM and (E) EDX map of single CoNiCuPdPt NP highlighting the core@shell structure. (F) Experimental radial profiles of each element within the NP. Cobalt signals (Co Kα) are displayed in green, nickel signals (Ni Kα) are displayed in pink, copper signals (Cu Kα) are displayed in red, palladium signals (Pd Kα) are displayed in blue, and platinum signals (Pt Lα) are displayed in purple.

Analogous to the bimetallic systems, rapid addition of each of the 5 metal precursors (10,000 nmol/s) resulted in the formation of core@shell NPs. Low magnification HAADF-STEM and EDX images (Figure 6A-B) show pseudospherical core@shell NPs, averaging 12.9 ± 2.5 nm in diameter (Figure S22). Compositional analyses from both ensemble (ICP-OES, Figure 6C) and single particle (EDX, Table S8) measurements confirm the presence of all 5 elements in the final NP samples, although both the Co and Ni incorporation deviated from the targeted equimolar stoichiometry (*vide infra*). High magnification HAADF-STEM images (Figures 6D, S23) support the core@shell assignment, evidenced by the contrast between the core and shell regions and supported by radial profile analysis (Figure 6E,F). Here, we observed the core comprised of a Pd rich PdCu alloy, while Pt, Co, and Ni were localized in the shell. This chemical ordering is consistent with sequential reduction of the precursors based on their estimated reduction rates (Figure S24).

**Figure 7:**
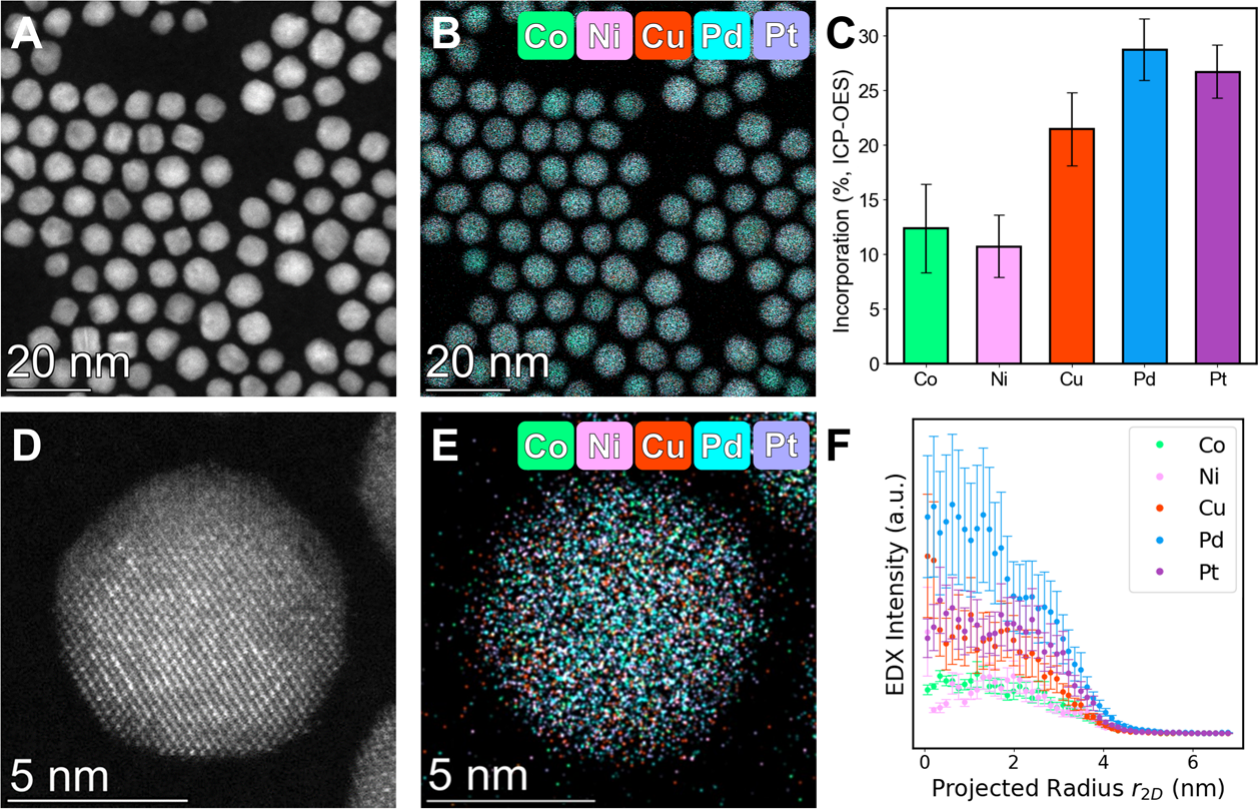
Structural characterization of 5 component CoNiCuPdPt NPs produced using a slow addition rate of 2.78 nmol/s. (A) Low magnification HAADF-STEM image and (B) corresponding EDX map of CoNiCuPdPt NPs. (C) Bar graphs of the incorporation of each metal as measured by ICP-OES. (D)High magnification HAADF-STEM image showing a single crystal NP and representative of crystal structures observed in these NPs (Figure S25). (E) EDX map of single CoNiCuPdPt NPs indicating their alloyed structure. (F) Experimental radial profiles of each element within the NP. Cobalt signals (Co Kα) are displayed in green, nickel signals (Ni Kα) are displayed in pink, copper signals (Cu Kα) are displayed in red, palladium signals (Pd Kα) are displayed in blue, and platinum signals (Pt Lα) are displayed in purple.

On the other hand, when the 5 metal precursors were titrated into the reaction using a slow addition rate (2.78 nmol/s of each precursor) alloyed NPs were observed. Low magnification HAADF-STEM and EDX images (Figure 7A,B) show the alloyed structure and reveal particles with an average diameter of 7.7 ± 1.1 nm (Figure S22). Compositional analyses from both ensemble (ICP-OES, Figure 7C) and single particle (EDX, Table S8) measurements confirm the presence of all 5 elements in the final NP samples. High magnification HAADF-STEM images (Figures 7D, S25) support the alloy assignment, with radial profile analysis (Figure 7E,F) revealing all 5 elements distributed evenly throughout the NP.

**Figure 8:**
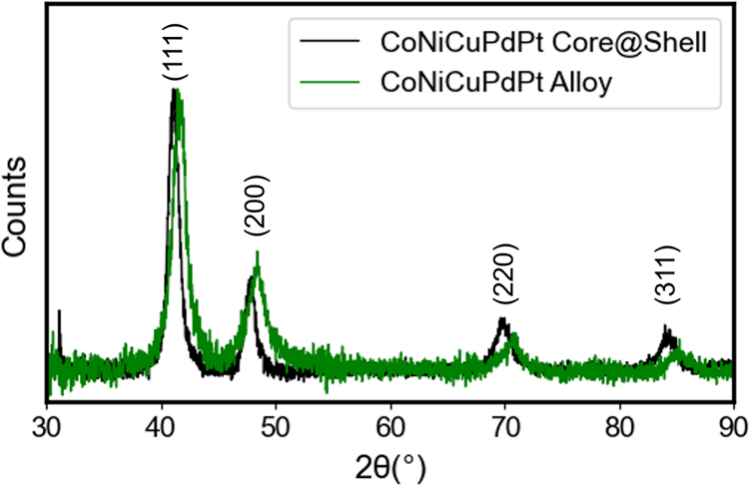
Experimental PXRD patterns obtained for 5 component CoNiCuPdPt NPs synthesized using the rapid (10,000 nmol/s, black trace) and slow (2.78 nmol/s, green trace) addition rates.

The difference in chemical ordering between the rapid and slow addition rates was further supported by PXRD analysis (Figure 8). Here, both addition rates produced populations consistent with an fcc crystal structure, however we observed a marked shift in both the peak positions and peak intensities between the two samples. The lattice parameters of the samples were estimated by fitting the experimental PXRD patterns (Figures S26, S27), and calculating the lattice constant using each peak (Tables S9 and S10). This analysis supported the assignment of an alloy phase produced using the slow addition rate, having a lattice parameter of 3.766 ± 0.001 Å, consistent with the expected lattice parameter based on the weighted average of the individual lattice constants of the constituent metals (Table S7).^73^ The observed decrease in relative peak intensity further corroborated the alloy assignment, where this decrease is commonly observed in high-entropy NP alloys and is attributed to displacements in atomic positions within the unit cell induced by differences in atomic radii between the constituent metals.^74^ We further characterized this decay by comparing the experimental pattern to a simulated PXRD pattern using the same crystallite size and lattice parameter, but assuming no off site displacements (Figure S28).

Notably, both addition rates produced NPs with the same stoichiometry within error and consistent with the observations and model described for the bimetallic cases above. This stoichiometric agreement extended even to Co and Ni content, which deviated from the targeted equimolar stoichiometry to the same degree in both the fast and slow addition cases. Similar deviations have been observed in other high-entropy syntheses using these metals, and are attributed to their slower reduction rate and greater oxophilicity.^21,75−77^ To understand if this deviation was the result of a kinetic factor, we performed a series of control experiments. First, we extended the reaction time at the same temperature to ensure the complete reduction of the Co and Ni precursors (Figure S29). However, the composition of this sample overlapped within error of the products obtained after 1 h of heating. Next, we increased the total amount of Co and Ni added to the synthesis. After increasing these amounts, we did observe an increase in the Co and Ni incorporation, however the final incorporation of both metals remained approximately 50% less than the amount added (Figure S30), suggesting that their limited incorporation is not due to a change in their concentration-controlled reaction rates. Instead, these results suggest the possibility of side reactions consuming some fraction of Co and Ni precursors during the synthesis (e.g., the formation of soluble oxides) and/or the leaching of the metallic Co and Ni from the NPs during the washing process.^21^

## Conclusions

In summary, we have used a quantitative model to describe the evolution of relative metal ion reduction rates as a function of precursor addition rate, which ultimately allows us to predict optimal addition rates for the synthesis of multimetallic nanoparticles with desired structural and compositional outcomes. Specifically, we observe that rapid addition rates allow the intrinsic reduction kinetics to drive particle outcomes and in the case of metal ions with large differences in intrinsic reduction rates, promotes the formation of core@shell architectures. Conversely, slow addition rates promote the synchronous reduction of precursors, facilitating alloy formation without sacrificing stoichiometric flexibility. Extending this strategy to a quinary (high-entropy) system comprised of Co, Ni, Cu, Pd, and Pt, we achieve similar control over nanoparticle stoichiometry and atomic arrangement, demonstrating the method’s potential utility for the synthesis of complex multicomponent systems. Taken together, these findings suggest that metal arrangement outcomes can be tuned by manipulating the precursor addition rate to control the relative reduction kinetics, and can remove the need to develop alternative metal ion precursors or reducing agent chemistries often required in the synthesis of complex metal NP architectures.

## Experimental Section

### Materials and Chemicals

Tetrachloroauric acid (HAuCl_4_), tetrachloropalladic acid (H_2_PdCl_4_), platinum tetrachloride (PtCl_4_), palladium(II) acetylacetonate (Pd(acac)_2_), platinum(II) acetylacetonate (Pt(acac)_2_), copper(II) acetylacetonate (Cu(acac)_2_), cobalt(II) acetylacetonate (Co(acac)_2_), Ni (II) acetylacetonate (Ni(acac)_2_), and technical grade oleylamine (OAm, 70%) were purchased from Sigma-Aldrich. 1,2-hexadecanediol (HDD, 98%) was purchased from TCI Chemicals. Absolute ethanol (EtOH), toluene, and chloroform (CHCl_3_) were purchased from Thermo Fisher Scientific (Pittsburgh, PA). All chemicals were used as received with no additional purification unless otherwise noted. All syntheses were carried out under argon using standard Schlenk-line techniques. Post-synthetic purifications were carried out in air. Prior to use, all glassware and Teflon stir bars were washed in aqua regia and rinsed with copious amounts of water prior to oven drying. *Caution*: *Aqua regia is highly toxic and corrosive and should only be used with proper personal protective equipment and training. Aqua regia should be handled inside a fume hood only.*

### Synthesis of Au–Pd Bimetallic Nanoparticles

Both core@shell and alloyed NPs were obtained using the same procedure by varying the rate of metal precursor addition. For the synthesis of Au@Pd NPs, the metal ion precursor solution is first made by adding HAuCl_4_ (1 μmol) and H_2_PdCl_4_ (1 μmol) to a dry 2-neck round-bottom flask. Then, 1 mL of OAm is added to the round-bottom, and the resulting mixture is kept at room temperature and under vacuum for 1 h before refilling with Ar. Separately, 9 mL of OAm and 0.5 mmol of HDD (131 mg) were added to a 3-neck round-bottom flask and degassed at 90 °C for 1 h. Afterward, the OAm/HDD solution was heated to 140 °C under Ar. When the solution reached 140 °C, the metal precursor solution was removed from its flask via syringe and quickly injected into the OAm/HDD mixture. Temperature was monitored by a temperature controller (Cole-Parmer) and a corresponding, glass-enclosed, temperature probe placed directly into the solution via one of the three necks. After injection of metal precursors, the reaction mixture was maintained at 140 °C for 1 h, before removing from the heating mantle and allowing to cool to 60 °C. Once the reaction mixture reached 60 °C, the contents of the reaction flask were then divided into 2, 50 mL centrifuge tubes (Corning) along with 30 mL of EtOH followed by centrifugation at 16,639 rcf for 10 min. After two washing cycles, the NPs were resuspended in 500 μL of toluene for further characterization.

For the synthesis of alloyed AuPd NPs, 10 mL of an OAm solution containing HAuCl_4_ and H_2_PdCl_4_ (1 μmol each) was added into the reaction flask at a rate of 10 mL/hour (or 0.2 nmol/s). After the precursor solution had been added, the reaction mixture was maintained at 140 °C for an additional 10 min, before cooling to 60 °C. The particles were isolated and washed using the same procedure described above, before storing in toluene for further characterization.

### Synthesis of Au–Pt Bimetallic Nanoparticles

The synthesis of AuPt NPs uses the same procedure as described above, except H_2_PdCl_4_ is replaced with the platinum precursor, PtCl_4_, and the reaction temperature is increased from 140 to 160 °C to accommodate for the lower reactivity of the platinum precursor. Briefly, HAuCl_4_ (1 μmol) and PtCl_4_ (1 μmol) were added to a 2-neck round-bottom flask with either 1 mL (for the synthesis of core@shell NPs) or 12 mL (for the synthesis of alloy NPs) of OAm and kept under vacuum for 1 h before refilling with Ar. Separately, 9 mL of OAm and 0.5 mmol of HDD (131 mg) were added to a 3-neck round-bottom flask and degassed at 90 °C for 1 h. Afterward, the OAm/HDD solution was heated to 160 °C under Ar, at which point the metal precursor solution was added into the reaction flask. The solution was then maintained at 160 °C for 1 h, before removing from the heating mantle and cooling to 60 °C. After reaching 60 °C, NPs were isolated and washed using the same procedure described above, before storing in toluene for further characterization.

### Synthesis of CoNiCuPdPt Quinary Nanoparticles

For the synthesis of CoNiCuPdPt NPs, 10 μmol each of Co(acac)_2_, Ni(acac)_2_, Cu(acac)_2_, Pd(acac)_2_, and Pt(acac)_2_ were added to a 2 neck round-bottom flask along with either 1 mL (for the synthesis of core@shell NPs) or 12 mL (for the synthesis of alloy NPs) of OAm and degassed at room temperature for 1 h. The solution was then stirred at 60 °C under Ar for 20 min until dissolved, before cooling again to room temperature. Separately, 9 mL of OAm and 0.5 mmol of HDD (131 mg) were added to a 3-neck round-bottom flask and degassed at 110 °C for 1 h. Afterward, the OAm/HDD solution was heated to 220 °C under Ar, at which point the metal precursor solution was added into the reaction flask. The solution was maintained at 220 °C for 1 h, before removing from the heating mantle and allowing to cool to 60 °C. The particles were isolated and washed using the same procedure described above, before storing in toluene for further characterization. We note that for the synthesis of quinary NPs, we chose to use acetylacetonate precursors. Although the acetylacetonate anion could act as a source of oxygen in our reaction, we found it necessary due to the limited solubility of the Ni and Cu chloride salts, as well as possible disproportion reactions of several of the chloride precursors in oleylamine.^78−80^

### Measurement of Reduction Rate Constants

The reduction rates of the precursors were determined by monitoring the reaction progress via UV–vis extinction spectroscopy (Cary 5000 UV–vis-NIR, Agilent, *vide infra*) as well as by tracking the metal ion concentration in the reaction solution at different time points using inductively coupled plasma optical emission spectrometry (ICP-OES, PerkinElmer, *vide infra*). These two methods are well-established for determining metal ion reduction rates, and were both used here for additional analytical clarity.^19,20,24,28^ Here, 1 mL of precursor solution containing 10 μmol of each of the specific metal precursors was injected into the reaction flask under identical synthetic conditions used for the NP synthesis. Aliquots (100 μL) were taken at different time points and immediately injected into an Eppendorf tube with 900 μL of cold hexanes (0 °C), for UV–vis analysis, or into cold ethanol (0 °C) for ICP-OES analysis. For ICP-OES analysis, the solutions were then centrifuged in an Eppendorf 5424 centrifuge with a fixed angle rotor (F-45-30-11, Eppendorf) at 21,130 rcf for 10 min to precipitate the NPs. Then, the supernatant was removed, and the pellet was resuspended in minimal CHCl_3_ (250 μL) followed by the addition of 1000 μL of EtOH before additional centrifugation. This washing procedure was repeated 5 times. The concentrations of metal ions obtained at different time points were then used to determine the rate constant (*k*) by performing a linear fit to the plot of ln[Pd(II)] or ln[Pt(IV)] vs reaction time, with the slope of the regression line taken as −*k* (Figures S1–S3, Table S1).

### Characterization

#### High-Angle Annular Dark-Field Scanning Transmission Electron Microscopy (HAADF-STEM) and Energy Dispersive X-ray (EDX) Spectroscopy

All samples were prepared for TEM by drop casting an aliquot of purified solution onto carbon film-coated 200 mesh nickel (for the bimetallic samples) or molybdenum (for the quinary samples) TEM grids (Ted Pella, Inc., Redding, CA). To remove excess ligand and other hydrocarbon contamination, the TEM grids were treated with activated carbon, as described by Bals et al.^81^ Briefly, in a 100 mL beaker, granulated activated carbon (1 g) was mixed with 50 mL of EtOH. While the solution was still bubbling, the TEM grid was submerged for 2 min, before removing from the solution and gently wicking the excess ethanol away with a Kimwipe. The TEM grids were then stored under vacuum for at least 24 h before analysis.

The collection of HAADF-STEM and EDX images was performed on a Thermo Fisher Titan Themis Cs-corrected microscope at an accelerating voltage of 200 kV (Nanoscale Fabrication and Characterization Facility, Petersen Institute of Nanoscience and Engineering, Pittsburgh, PA). Velox 3.81 was used for drift correction during acquisition. The size distributions of the NPs were determined from HAADF-STEM images of at least 250 NPs from various areas of the grid, per experimental replicate, using ImageJ 1.53k (National Institutes of Health,), and at least 3 experimental replicates were combined to form the reported values. EDX data were acquired using 4k channels from 0 to 20 keV with a 5 eV dispersion. All images were collected with a dwell time of 3 μs/pixel. A pixel size of 92 pm/pixel was used for the bimetallic samples and a pixel size of 45 pm/pixel for the quinary samples. Standard Cliff-Lorimer (K-factor) quantification was used for quantification of EDX data (reported in atomic percent). Radial profile analyses were performed by adapting a method described in the literature and are treated in detail in the Supporting Information (eqs S1–S5 and associated discussion regarding construction of fitting equations and error estimation).^68^ Briefly, single particle EDX images are processed using a home-built python code to define the pixel coordinate of the origin for each NP. The image is then radially processed from this point to average the intensities of the EDX signal for each element, generating the radial profiles. Individual radial profiles were then averaged to generate the values and error bars shown in the main text and used for fitting to equations reported in the literature (eqs S1–S3).

#### Inductively Coupled Plasma Optical Emission Spectroscopy (ICP-OES)

ICP-OES analysis was performed using an argon flow with an Optima spectrometer (PerkinElmer, Inc.). An aqua regia solution was prepared with a 3:1 ratio of hydrochloric acid:nitric acid (Sigma-Aldrich, > 99.999% trace metal basis), a portion of which was diluted with NANOpure water for a 5% v/v aqua regia matrix. Purified NP samples were digested with ∼200 μL of ultrapure, concentrated aqua regia in a 10 mL volumetric flask and diluted to volume with the 5% aqua regia solution. The unknown metal concentrations were determined by comparison to a 7-point standard calibration curve with a range of 0.1–10 ppm prepared from standards for ICP (Fluka, TraceCERT 1000 ± 2 mg/L Co in HNO_3_, Fluka, TraceCERT 1000 ± 2 mg/L Ni in HNO_3_, Fluka, TraceCERT 1000 ± 2 mg/L Cu in HNO_3_, Fluka, TraceCERT 1001 ± 2 mg/L Pd in HCl, Fluka, TraceCERT 1000 ± 2 mg/L Pt in HNO_3_, Fluka, TraceCERT 1001 ± 2 mg/L Au in HCl), respectively, and diluted in the 5% aqua regia matrix. All standards and unknown samples were measured 3 times and averaged.

#### Powder X-ray Diffraction (PXRD)

PXRD data were collected on a Bruker D8 Discover XRD (Nanoscale Fabrication and Characterization Facility, Petersen Institute of Nanoscience and Engineering, Pittsburgh, PA) at 40 kV, 40 mA, with Cu Kα radiation (λ = 1.54 Å) using a step size of 0.02° and a scan speed of 1 s/step. The samples were prepared by drop casting NPs onto a zero-diffraction holder (MTI).

## Supporting Information Available

Additional data and methodology details including: simulated instantaneous concentration and reduction profiles for Au^3+^, Pd^2+^, and Pt^4+^; radial profile analysis and quantification, additional HAADF-STEM and EDX images of all NP samples, NP size distributions, additional PXRD patterns and analysis, reduction rate data, and additional analysis of control experiments (PDF)
