# Supplementary material for: Decoupling Intrinsic Metal Ion Reduction Rates from Structural Outcomes in Multimetallic Nanoparticles
Source: J Am Chem Soc. 2024 Dec 9;146(50):34822–32. doi: 10.1021/jacs.4c13826 (PMC11664505; doi:10.1021/jacs.4c13826)
Supplement: Supplementary file 1 — ja4c13826_si_001.pdf [file ja4c13826_si_001.pdf]

Supporting Information

Decoupling intrinsic metal ion reduction rates from structural outcomes in multimetallic nanoparticles

Jacob H. Smith[†], Qi Luo[†], Shelby L. Millheim[†], and Jill E. Millstone[†‡Δ]*

[†]Department of Chemistry, University of Pittsburgh, 219 Parkman Avenue, Pittsburgh, Pennsylvania 15260, United States

[‡]Department of Chemical and Petroleum Engineering, University of Pittsburgh, Pittsburgh, Pennsylvania 15260, United States

[Δ]Department of Mechanical Engineering and Materials Science, University of Pittsburgh, Pittsburgh, Pennsylvania 15260, United States

Table of Contents:

Figure S1. Determination of monometallic Pd^{2+} reduction rates. (A) Plots of the concentrations of Pd^{2+} ions in the reaction solution as a function of reaction time in both the absence (blue curves) and presence (green curves) of Au^{3+} (10 μmol). (B) Plots of ln[Pd^{2+}] as a function of time where fits assume pseudo first-order reaction kinetics. The slope of the fit is the reaction rate constant. 6

Figure S2. Determination of Au^{3+} reduction kinetics under our reaction conditions. (A) Extinction spectra of reaction solutions as a function of reaction time. (B) Plot of the normalized optical density (O.D.) of reaction solutions at λ_{max} (λ_{max} = 520 nm), where spectra are normalized to the peak intensity of the final reaction solution. AuNP formation was complete within 5 minutes. Inset: Photograph of aliquots from the reaction that have been quenched using cold hexanes. Color changes highlight the fast formation of Au NPs and their subsequent stability. (C) Plots of Au^{3+} ion concentrations in the reaction solution as a function of reaction time in the presence of Pd^{2+} (red curves) or Pt^{4+} (orange curves). (D) Plots of ln[Au^{3+}] as a function of time. Due to the fast reduction kinetics of the Au^{3+} ion in our system, we were unable to extract detailed kinetic data from these data. However, in conjunction with the UV-vis analysis, we were able to extrapolate a rate constant of 2.21 min^{-1}. 7

Figure S3. Determination of monometallic Pt^{4+} reduction rates. (A) Plots of the concentrations of Pt^{4+} ions in the reaction solution as a function of reaction time in the absence (purple curves) and presence (red curves) of Au^{3+} (10 μmol). (B) Plots of ln[Pt^{4+}] as a function of time where fits assume a pseudo-first-order reaction. The slope of the fit is the reaction rate constant. 7

Table S1. Measured rate constants for bimetallic systems. 7

Predicting Optimal Reduction Rates in Bimetallic Systems 7

Figure S4. Plots of the simulated instantaneous metal precursor concentration (A) and reduction rates (B) of Au^{3+} and Pd^{2+} when the metal precursors are added using the rapid addition rate of 1000 nmol/sec of each precursor. 9

Figure S5. Plots of the simulated instantaneous metal precursor concentration (A) and reduction rates (B) of Au^{3+} and Pd^{2+} when the metal precursors are added using the slow addition rate of 0.2 nmol/sec of each precursor. 9

Figure S6. Plot of the predicted relative amounts of Au^{3+} and Pt^{4+} reacted during a given metal addition period as a function of precursor addition rate. Each point represents a simulation corresponding to a different addition rate. The black circle (Point C, Pt:Au = 0.04) corresponds to a rapid addition rate of 1000 nmol/sec, per precursor and the red circle (Point D, Pt:Au = 0.86) corresponds to a slow addition rate of 0.2 nmol/sec, per precursor. 10

Figure S7. Low magnification STEM images and corresponding size histograms for (A,B) Au@Pd NPs synthesized using the 1000 nmol/sec addition rate and (C,D) AuPd alloy NPs synthesized using the 0.2 nmol/sec addition rate. 10

Figure S8. Additional HAADF-STEM image of the Au@Pd NPs produced using the 1000 nmol/sec addition rate, corresponding to Figure 2C in the main text and showing twinned crystal structures. 11

Radial Profile Analysis of EDX Images 12

Consideration of Electron Dose and its Possible Impact on NP Morphologies 13

Table S2. EDX Acquisition parameters used to estimate the electron dose delivered per frame (D_f). For bimetallic samples, a pixel size of 91 pm and a raster size of 1024x1024 pixels were used. For quinary samples, a pixel size of 45 pm and a raster size of 2048x2048 pixels were used. 13

Figure S9. HAADF-STEM images corresponding to the first and final frame of EDX map acquisition for (A, B) AuPd core@shell NPs and (C,D) AuPd alloy NPs. 14

Figure S10. HAADF-STEM images corresponding to the first and final frame of EDX map acquisition for (A, B) CoNiCuPdPt Core@Shell NPs and (C,D) CoNiCuPdPt alloy NPs. 14

Figure S11. Additional HAADF-STEM image of the AuPd alloy NPs produced using the 0.2 nmol/sec. 15

Figure S12. Extinction spectra of the Au@Pd NPs synthesized using the 1000 nmol/sec addition rate (red trace) and AuPd alloy NPs synthesized using the 0.2 nmol/sec addition rate (green trace). 16

Figure S13. Experimental PXRD pattern of Au@Pd NPs (black trace) and corresponding fit (red trace) used to determine peak positions. The residuals from the fit are shown as the blue trace. 16

Table S3. Optimized peak positions and calculated lattice parameters of Au@Pd NPs from the PXRD pattern shown in Figure S13 17

Figure S14. Experimental PXRD pattern of AuPd alloy NPs (black trace) and corresponding fit (red trace) used to determine the peak positions. The residuals from the fit are shown as the blue trace. 17

Table S4. Optimized peak positions and calculated lattice parameters of the AuPd alloy NPs from the PXRD pattern shown in Figure S14. 18

Figure S15. Overlay of PXRD patterns from Figures S13 and S14, where Au@Pd (red) and AuPd alloy NPs (green trace) are shown together. Due to the peak broadening induced by the crystallite size, visualizing the shift in the diffraction pattern is aided by this representation. 18

Figure S16. EDX images of Au@Pd NPs synthesized using an addition rate of 1000 nmol/sec followed by annealing at 220°C for six hours. Images indicate evolution in Pd shell faceting, but the core@shell structure is preserved in all cases and no evidence of alloying was observed..... 19

Figure S17. Structural characterization of bimetallic Au-Pt NPs produced using the rapid (1000 nmol/sec, A-E) and slow (0.2 nmol/sec, F-J) addition rates. (A) Low magnification HAADF-STEM image and (B) corresponding EDX map of Au@Pt NPs produced using a rapid precursor addition rate of 1000 nmol/sec. (C) EDX map of single Au@Pt core@shell NP (D) Experimental radial profile analysis of each element within the NP, along with the analytical fit to the data (solid lines). (E) Optimized parameters derived from the fit, supporting the core@shell assignment. (F) Low magnification HAADF-STEM image and (G) corresponding EDX map of AuPt alloy NPs produced using the slow addition rate of 0.2 nmol/sec. (H) EDX map of single AuPt alloy NP. (I) Experimental radial profile analysis of each element within the NP, along with the analytical fit to the data (solid lines). (J) Optimized parameters derived from the fit, supporting the alloy assignment. Platinum (Pt Lα) signals are displayed in purple and gold (Au Lα) signals in orange. 19

Figure S18. Size histograms for (A) Au@Pt NPs synthesized using the 1000 nmol/sec addition rate and (B) AuPt alloy NPs synthesized using the 0.2 nmol/sec addition rate. 20

Figure S19. (A-C) Plots of the simulated instantaneous metal precursor concentrations and (D-F) corresponding reduction rates of Au^{3+} and Pd^{2+} when the metal precursors are added using the slow precursor addition rate (0.4 nmol/sec, total precursor). Here, we predict that changing the initial molar ratio of Au^{3+} to Pd^{2+} will impact the relative instantaneous ion concentrations, and therefore the instantaneous reduction rates accordingly, while still facilitating their synchronous reduction. 20

Figure S20. EDX maps of AuPt NPs produced with different stoichiometries of Au:Pt using a (A-C) rapid addition rate (2,000 nmol/sec, total precursor) and (D-F) slow addition rate (0.4 nmol/sec, total precursor). Platinum (Pt Lα) signals are displayed in purple and gold (Au Lα) signals in orange. 21

Figure S21. Percent Au incorporated into the Au-Pt NP samples as a function of the initial molar percent Au added to the synthesis for both the rapid (circles) and slow (triangles) addition rates. The dashed line represents the theoretical stoichiometry assuming 1:1 incorporation. As in the case of the Au-Pd particles, nominal and incorporated stoichiometries agree well for both the core@shell and alloyed NPs. 22

Table S5. Composition outcomes for Au-Pd NPs synthesized using either rapid (2,000 nmol/sec, total precursor) or slow (0.4 nmol/sec, total precursor) addition rates as determined by both single particle (EDX) and ensemble (ICP-OES) analyses. 22

Table S6. Composition outcomes for Au-Pt NPs synthesized using either rapid (2,000 nmol/sec, total precursor) or slow (0.4 nmol/sec, total precursor) addition rates as determined by both single particle (EDX) and ensemble (ICP-OES) analyses. 23

Table S7. Physicochemical Properties of Metals in the Quinary System 23

Figure S22. Low magnification STEM images and corresponding size histograms for (A, B) CoNiCuPdPt core@shell NPs synthesized using a rapid addition rate and (C, D) CoNiCuPdPt alloyed NPs synthesized using a slow addition rate. 24

Table S8. Composition of quinary NPs synthesized using both rapid and slow addition rates as determined by both single particle (EDX) and ensemble (ICP) analyses. 24

Figure S23. Additional HAADF-STEM image of the CoNiCuPdPt NPs produced using the rapid addition rate of 10,000 nmol/sec. 25

Figure S24. Analysis of the relative reduction kinetics in the synthesis of quinary CoNiCuPdPt NPs. Plots of ln[M^{2+}] (M = Co, Ni, Cu, Pd, or Pt) as a function of time with fits assuming pseudo first-order reaction kinetics (A). Here, the relative reduction kinetics we observe (Pd > Cu > Pt > Co, Ni) are consistent with the chemical arrangement observed in the core@shell structures produced using a rapid addition rate. (B) Plot of the predicted amounts of Co^{2+}, Ni^{2+}, Cu^{2+} or Pt^{2+} reacted relative to the amount of Pd^{2+} reacted during a given metal addition period as a function of precursor addition rate and used to predict the addition rate for alloy production. 26

Figure S25. Additional HAADF-STEM image of the CoNiCuPdPt NPs produced using the slow addition rate of 2.27 nmol/sec, per precursor. 27

Figure S26. Experimental PXRD pattern of core@shell CoNiCuPdPt NPs synthesized using the rapid addition rate of 10,000 nmol/sec (black trace) and corresponding fit (red trace) used to determine the peak positions. The residuals from the fit are shown as the blue trace. 28

Table S9. Optimized peak positions and calculated lattice parameters from the PXRD fit of the core@shell CoNiCuPdPt NPs shown in Figure S26 synthesized using the rapid addition rate of 10,000 nmol/sec. 28

Figure S27. Experimental PXRD pattern of alloyed CoNiCuPdPt NPs synthesized using the slow addition rate of 2.27 nmol/sec (black trace) and corresponding fit (red trace) used to determine the peak positions. The residuals from the fit are shown as the blue trace. 29

Table S10. Optimized peak positions and calculated lattice parameters from the PXRD fit of pattern shown in Figure S27 from CoNiCuPdPt NPs synthesized using the slow addition rate of 2.27 nmol/sec. 29

Figure S28. Experimental PXRD pattern of alloy CoNiCuPdPt NPs (black trace) compared to a simulated pattern assuming no off-site displacements (red trace). Simulated pattern was generated using Crystal Maker® version 11.0.2. 30

Figure S29. HAADF-STEM and corresponding EDX maps for CoNiCuPdPt core@shell NPs after annealing at the reaction temperature for 2 additional hours. Here, increasing the reaction time did not increase the incorporation of Co and Ni (incorporation determined by area EDX analysis). 30

References 31

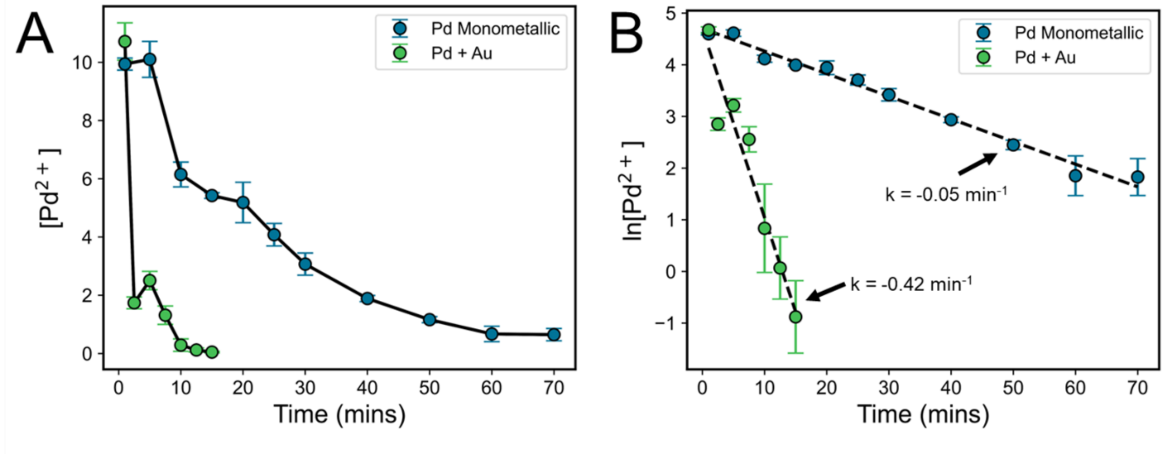

Figure S1. Determination of monometallic Pd^{2+} reduction rates. (A) Plots of the concentrations of Pd^{2+} ions in the reaction solution as a function of reaction time in both the absence (blue curves) and presence (green curves) of Au^{3+} (10 µmol). (B) Plots of ln[Pd^{2+}] as a function of time where fits assume pseudo first-order reaction kinetics. The slope of the fit is the reaction rate constant.

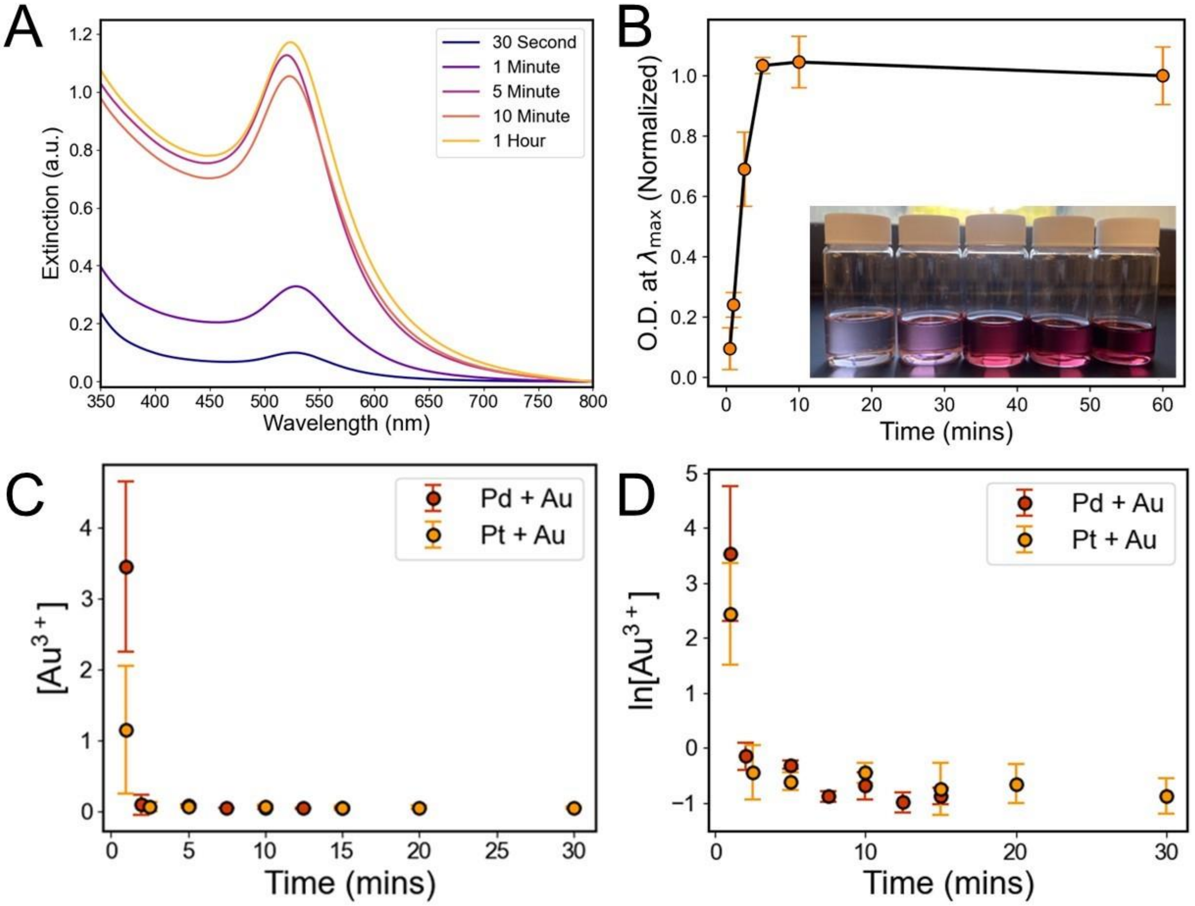

Figure S2. Determination of Au^{3+} reduction kinetics under our reaction conditions. (A) Extinction spectra of reaction solutions as a function of reaction time. (B) Plot of the normalized optical density (O.D.) of reaction solutions at λ_{max} (λ_{max} = 520 nm), where spectra are normalized to the peak intensity of the final reaction solution. AuNP formation was complete within 5 minutes. Inset: Photograph of aliquots from the reaction that have been quenched using cold hexanes. Color changes highlight the fast formation of Au NPs and their subsequent stability. (C) Plots of Au^{3+} ion concentrations in the reaction solution as a function of reaction time in the presence of Pd^{2+} (red curves) or Pt^{4+} (orange curves). (D) Plots of ln[Au^{3+}] as a function of time. Due to the fast reduction kinetics of the Au^{3+} ion in our system, we were unable to extract detailed kinetic data from these data. However, in conjunction with the UV-vis analysis, we were able to extrapolate a rate constant of 2.21 min^{-1}.

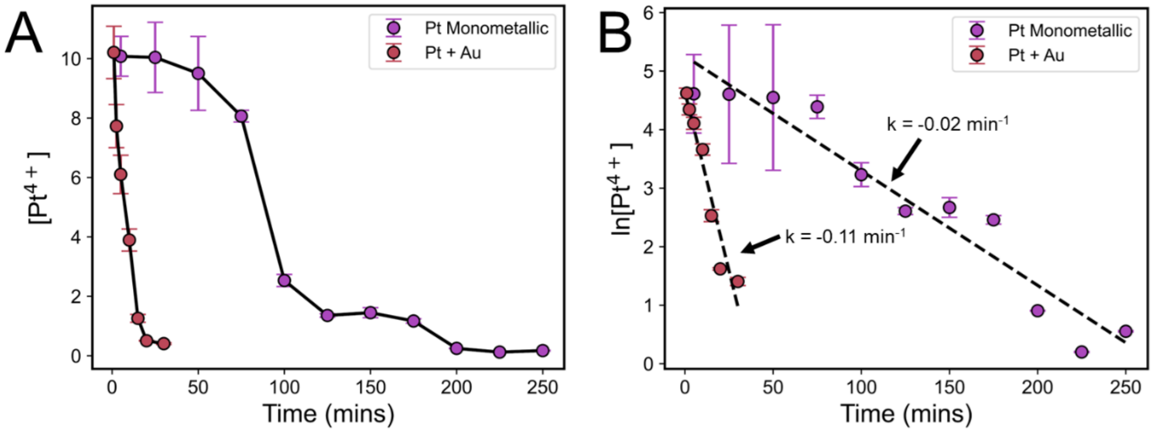

Figure S3. Determination of monometallic Pt^{4+} reduction rates. (A) Plots of the concentrations of Pt^{4+} ions in the reaction solution as a function of reaction time in the absence (purple curves) and presence (red curves) of Au^{3+} (10 μmol). (B) Plots of ln[Pt^{4+}] as a function of time where fits assume a pseudo-first-order reaction. The slope of the fit is the reaction rate constant.

Table S1. Measured rate constants for bimetallic systems.

System	k (min^{-1})
Au^{3+}	2.21
Pd^{2+}	0.42
Pt^{4+}	0.11

Predicting Optimal Reduction Rates in Bimetallic Systems

We determined reduction rate constants for the metal precursors in both the presence and absence of the secondary metal. At first glance, it would seem most straightforward to predict the optimal addition rates using the reduction rates of the monometallic cations alone. However, the presence of other metal ions or metal surfaces can significantly alter these rates.[1,2] Therefore, we used the rate constants obtained in the presence of the secondary metal to predict the change in metal ion

concentration as function of metal ion addition rate in our system. Using these reduction rate constants (Table S1) and equation 3, we can then simulate how the instantaneous metal ion concentrations and reduction rates evolve as a function of precursor addition rate, assuming a constant amount of metal precursor in all cases.

As described in the main text, to estimate whether a given addition rate would result in sequential or simultaneous reduction of the Au^{3+} and Pd^{2+} precursors, we calculated the relative amounts of Au^{3+} and Pd^{2+} reacted during the metal addition period by integrating equation 3 from t = 0 to $\frac{V_s}{r}$. These values are plotted for 100 different addition rates spanning 6 orders of magnitude and are shown in Figure 1 of the main text. In this graph, low ratios of reacted Pd^{2+}:Au^{3+} indicate that Pd^{2+} reduction lags behind Au^{3+} reduction, suggesting that addition rate is likely to produce core@shell NPs, since there will remain unreacted Pd^{2+} ions in solution after metal addition ceases. On the other hand, ratios of reacted precursor (Pd^{2+}:Au^{3+}) closer to one, indicate simultaneous reduction of Au^{3+} and Pd^{2+} and are more likely to produce alloyed NPs. This same approach was used to predict optimal addition rates for the AuPt system (Figure S6).

The specific addition rates used to produce core@shell and alloy NPs were selected by identifying addition rates within the two plateau regions of the function depicted in main text Figure 1. Specifically, we chose a rapid addition rate corresponding to 1000 nmol/sec of each precursor (black circle in Figure 1), and a slow addition corresponding to 0.2 nmol/sec of each precursor (red circle in Figure 1).

We note that in looking at the position of the red circle (0.2 nmol/sec) it is clear there are addition rates that could result in values closer to 1. However, practical considerations influenced our choice of the 0.2 nmol/sec addition rate. For example, reducing the addition rate by another order of magnitude, from 0.2 nmol/sec to 0.02 nmol/sec, would have extended the metal addition period to 10 hours. In our control experiments, reactions of this length required a droplet to be in the syringe and exposed to high temperatures for extended periods before being released into the reaction mixture. Under those conditions, metal ion precursor reduction began before the droplet was introduced to the reaction and therefore was no longer suitable for our analysis. Given these constraints, we chose the 0.2 nmol/sec addition rate, which corresponds to a metal addition period of 1 hour. Both theoretical predictions (*vide infra*, Figure S4) and experimental results (main text Figure 3) confirmed that this rate was sufficient to produce alloy structures with >95% yield.

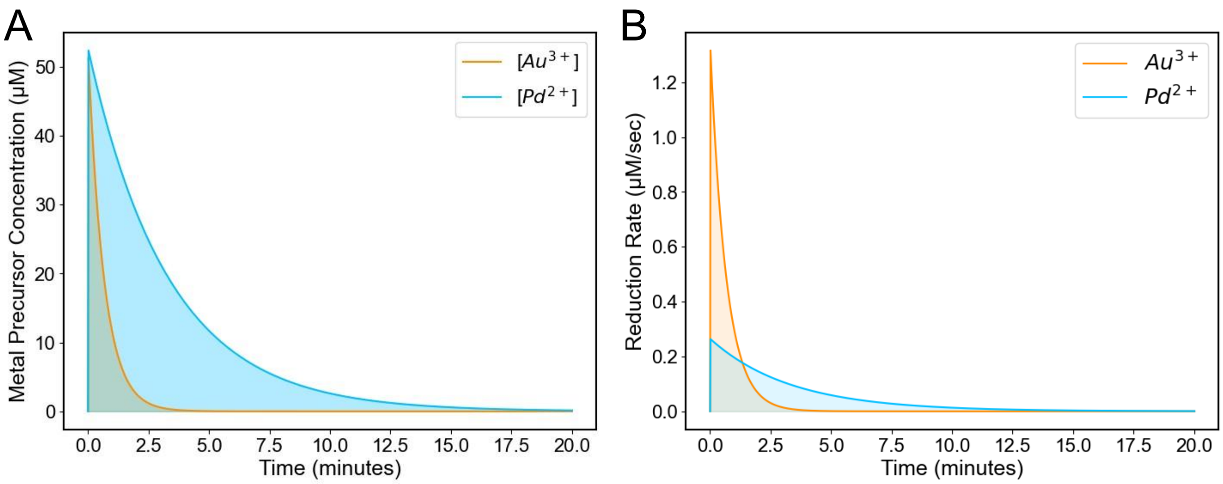

Figure S4. Plots of the simulated instantaneous metal precursor concentration (A) and reduction rates (B) of Au^{3+} and Pd^{2+} when the metal precursors are added using the rapid addition rate of 1000 nmol/sec of each precursor.

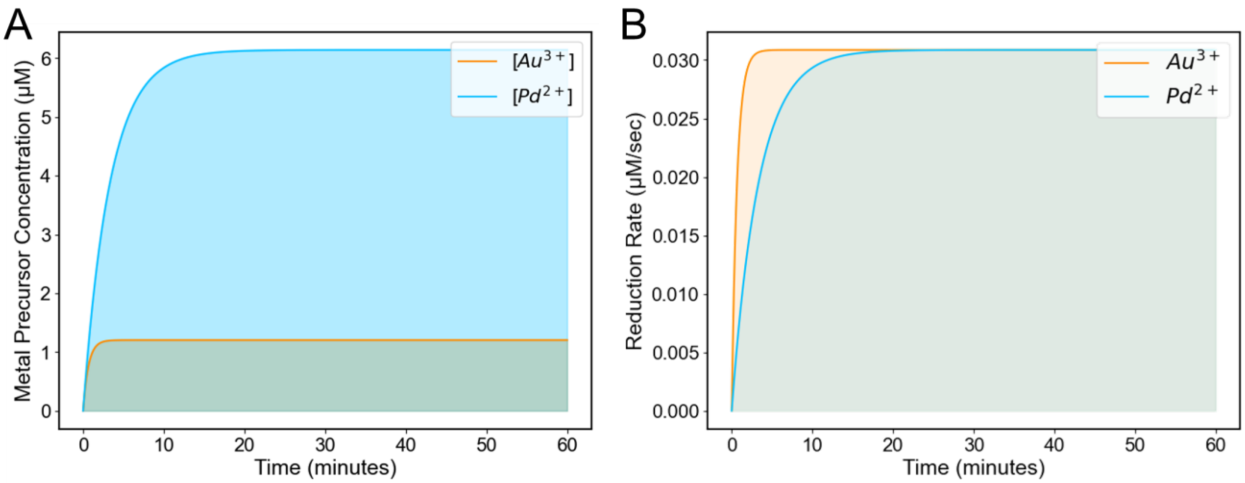

Figure S5. Plots of the simulated instantaneous metal precursor concentration (A) and reduction rates (B) of Au^{3+} and Pd^{2+} when the metal precursors are added using the slow addition rate of 0.2 nmol/sec of each precursor.

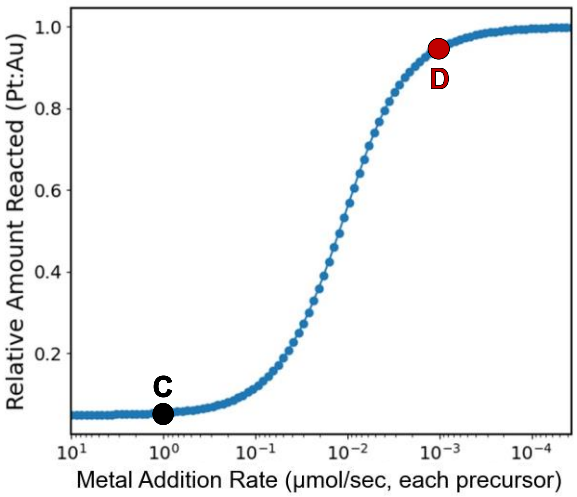

Figure S6. Plot of the predicted relative amounts of Au^{3+} and Pt^{4+} reacted during a given metal addition period as a function of precursor addition rate. Each point represents a simulation corresponding to a different addition rate. The black circle (Point C, Pt:Au = 0.04) corresponds to a rapid addition rate of 1000 nmol/sec, per precursor and the red circle (Point D, Pt:Au = 0.86) corresponds to a slow addition rate of 0.2 nmol/sec, per precursor.

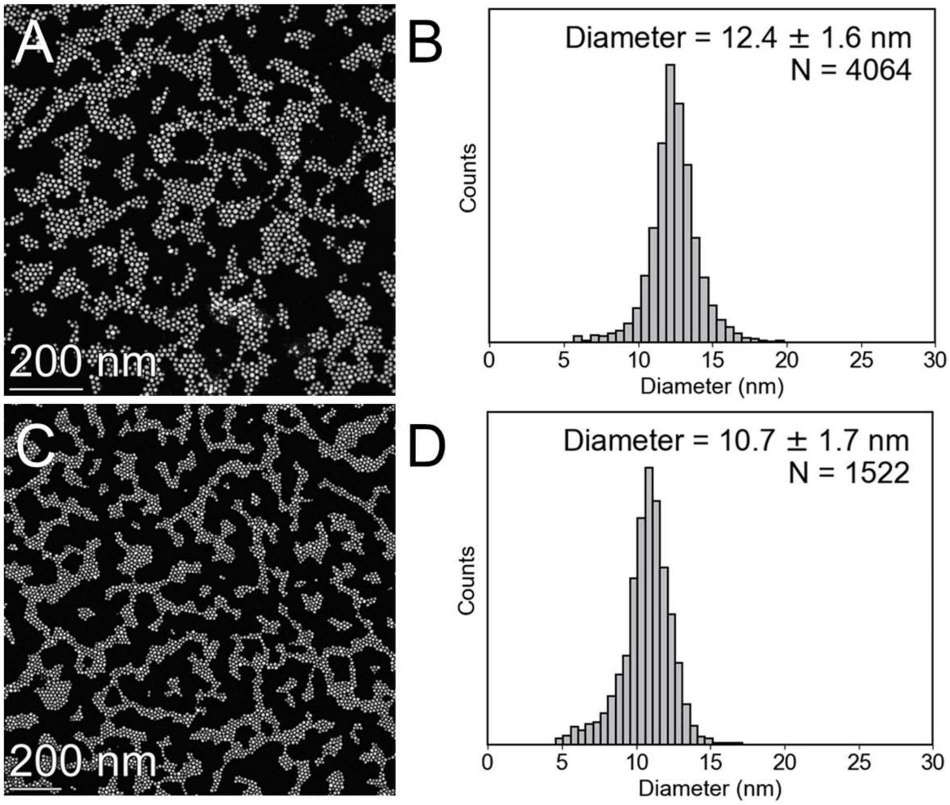

Figure S7. Low magnification STEM images and corresponding size histograms for (A,B) Au@Pd NPs synthesized using the 1000 nmol/sec addition rate and (C,D) AuPd alloy NPs synthesized using the 0.2 nmol/sec addition rate.

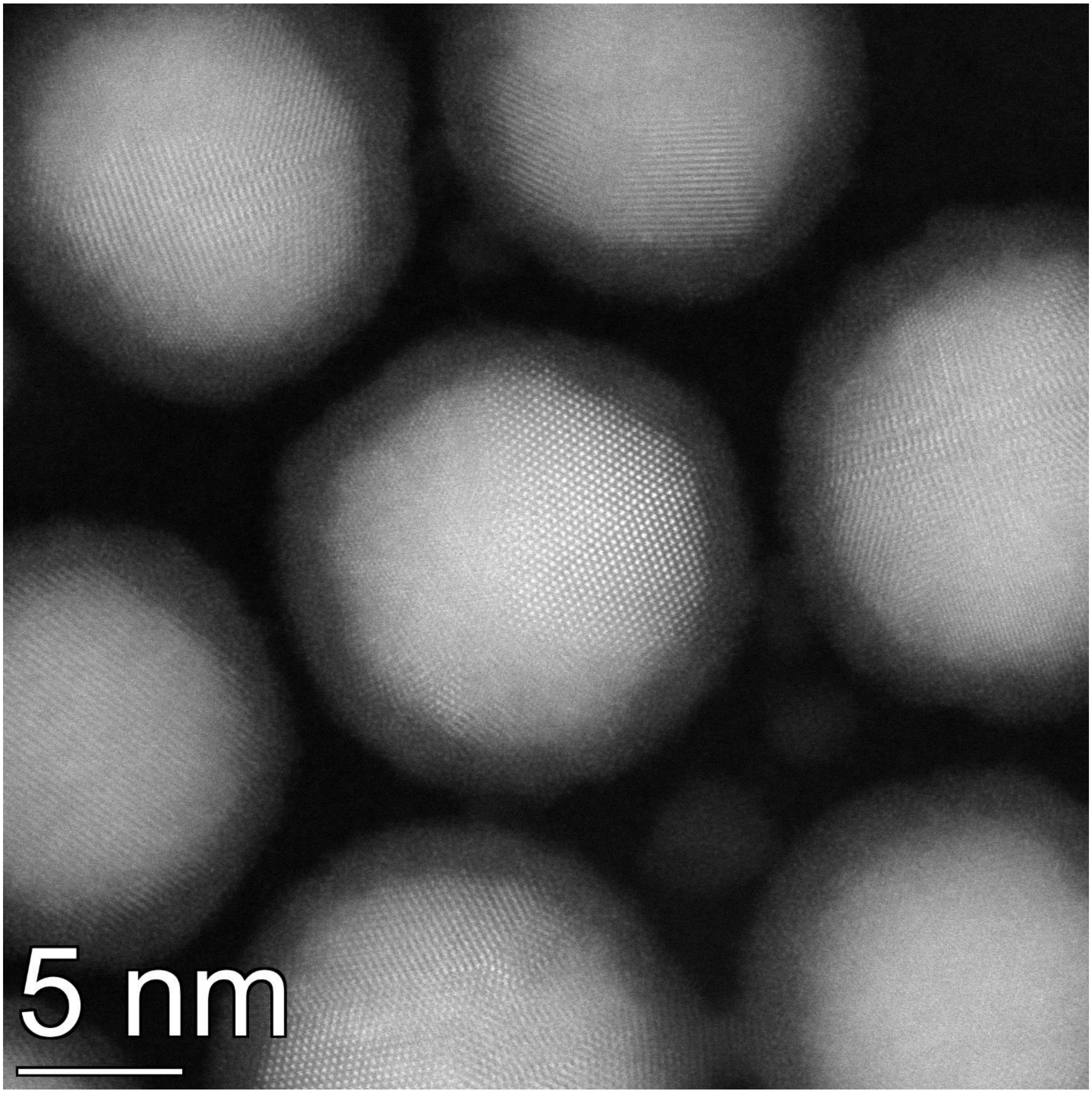

Figure S8. Additional HAADF-STEM image of the Au@Pd NPs produced using the 1000 nmol/sec addition rate, corresponding to Figure 2C in the main text and showing twinned crystal structures.

Radial Profile Analysis of EDX Images

As described in the main text, radial profiles of NP composition were generated by adapting a method described in the literature.[3] Using this method, two-dimensional (2D) EDX images are first geometrically transformed from cartesian to cylindrical coordinates using a home-built Python code to define the pixel coordinates corresponding to the center of each NP, excluding NPs having aspect ratios below 0.9 or above 1.1. The image is then processed radially from this point to average the EDX signal intensities for each element, allowing the EDX intensities to be expressed as one-dimensional (1D) functions of the projected radius (r_{2D}). Individual radial profiles were then averaged to generate the values and error bars shown in the main text. We chose to represent the EDX data using radial profiles, as opposed to other common approaches such as linescans, because the (pseudo-)spherical geometry of our NPs, particularly the core@shell chemical arrangement, is more effectively visualized in the cylindrical coordinate system.

In the case of bimetallic core@shell NPs, structural parameters were extracted from these data by fitting the experimental data to equations reported in the literature procedure (equations S1 and S2) using the Nelder-Mead optimization method.[3]

$$C_{core} = \frac{1}{2}\left(1 - erf\left(\frac{R - R_{core}}{\sqrt{2}\sigma_i}\right)\right) + \rho\left(\left(\frac{1}{2}\left(1 - erf\left(\frac{R - R_{shell}}{\sqrt{2}\sigma_s}\right)\right)\right) - \frac{1}{2}\left(1 - erf\left(\frac{R - R_{core}}{\sqrt{2}\sigma_i}\right)\right)\right) \quad (S1)$$

$$C_{shell} = (1 - \rho)\left(\left(\frac{1}{2}\left(1 - erf\left(\frac{R - R_{shell}}{\sqrt{2}\sigma_s}\right)\right)\right) - \frac{1}{2}\left(1 - erf\left(\frac{R - R_{core}}{\sqrt{2}\sigma_i}\right)\right)\right) \quad (S2)$$

Here, the fractional concentrations of the core metal (m_1) and shell metal (m_2) are defined in spherical coordinates as a function of the spherical radius (R, nm), the radius of the core R_{core} (nm), the radius of the shell R_{shell} (nm), the core@shell interface σ_i (nm), and the surface roughness σ_s (nm). The ρ term then describes the amount of core material present in the shell. To account for the fact that the EDX maps are 2D projections of 3D objects, the fitting functions are constructed in cylindrical coordinates (radial coordinate r_{2D} and axial coordinate z, where $R^2_{spherical} = r^2_{2D} + z^2$). During the fitting process, the equations are integrated with respect to the z-axis (corresponding to the STEM beam direction).

To analyze the alloyed NP data, a different approach was required. Here, the radial profile data of each element were fit independently using equation S3.

$$C_{m1,m2} = \frac{1}{2}\left(1 - erf\left(\frac{R - R_{m1,m2}}{\sqrt{2}\sigma_i}\right)\right) \quad (S3)$$

In this equation, $R_{m1,\,m2}$ represents the determined radius corresponding to m_1 and m_2. In the case of an alloy, this value will be the same for both components, whereas in a core@shell structure, the determined radii will be different. We used this analysis to support the alloy assignment of

the AuPd NPs produced using the slow addition rate (Figure 3) and found the radii of both Au and Pd to overlap within error of the estimated parameters.

To obtain error estimates for the fitted parameters, we also followed a procedure described in the literature.[4,5] Briefly, we first fit our experimental data to the fitting functions (equations S1-S3) by minimizing the quantity:

$$\chi^2 = \sum_i r_{2D_i} (y_i^{exp} - y_i^{fit})^2 \qquad (S4)$$

where r_{2D_i} is the radius associated with the ith data point, included as a weighting factor to account for the increasing number of pixels at larger radii, and y_i^{exp} and y_i^{fit} are the experimental and fitted intensities, respectively. The errors associated with the optimized parameters are then estimated by constructing a quadratic approximation of the error surface near the optimized parameters.[4]

Consideration of Electron Dose and its Possible Impact on NP Morphologies

To understand the extent of electron beam induced damage to our samples, we estimated the electron dose delivered during EDX acquisition using the parameters reported in Table S2 and equation S5, where I_b is the beam current, A is the acquisition area, and t_f is the time to acquire one frame.[6–9]

Table S2. EDX Acquisition parameters used to estimate the electron dose delivered per frame (D_f). For bimetallic samples, a pixel size of 91 pm and a raster size of 1024x1024 pixels were used. For quinary samples, a pixel size of 45 pm and a raster size of 2048x2048 pixels were used.

Sample	Current (pA)	Dwell Time (µS)	Aquisiton Area (nm^2)	Pixel Size (pm)	Raster Size (pixels)	D_f (e$^-$/Å^2)
Bimetallic	157	3	8772	91.47	1024×1024	3.51×10^3
Quinary	157	3	8772	45.73	2048×2048	1.41×10^4

$$D_f = \left(\frac{I_b}{A}\right)(t_f) \qquad (S5)$$

We also compared the HAADF-STEM images corresponding to the initial and final frames of an EDX acquisition for the bimetallic Au-Pd and quinary systems (Figures S9 and S10, corresponding to the EDX images in the main text). In the Au-Pd system, we observe a blurring

around the edges of the NPs indicating some surface reconstruction, and it is more pronounced in the Au@Pd NPs than in the AuPd alloyed NPs. The extent of this damage is consistent with the relatively large electron doses delivered during EDX acquisition and reflects the composition of our samples, with the AuPd alloy NPs exhibiting greater stability compared to the Au@Pd samples.[9,10] In all cases, the overall metal ordering in the particle does not change for any system under these conditions.

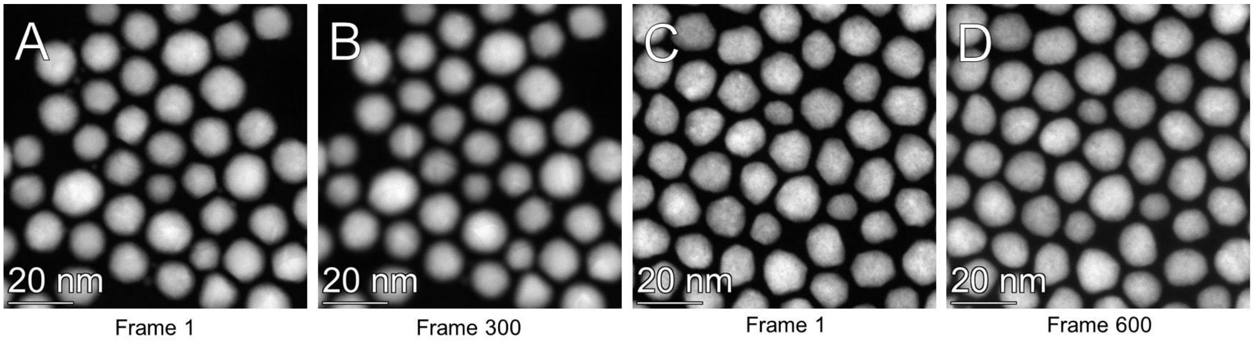

Figure S9. HAADF-STEM images corresponding to the first and final frame of EDX map acquisition for (A, B) AuPd core@shell NPs and (C,D) AuPd alloy NPs.

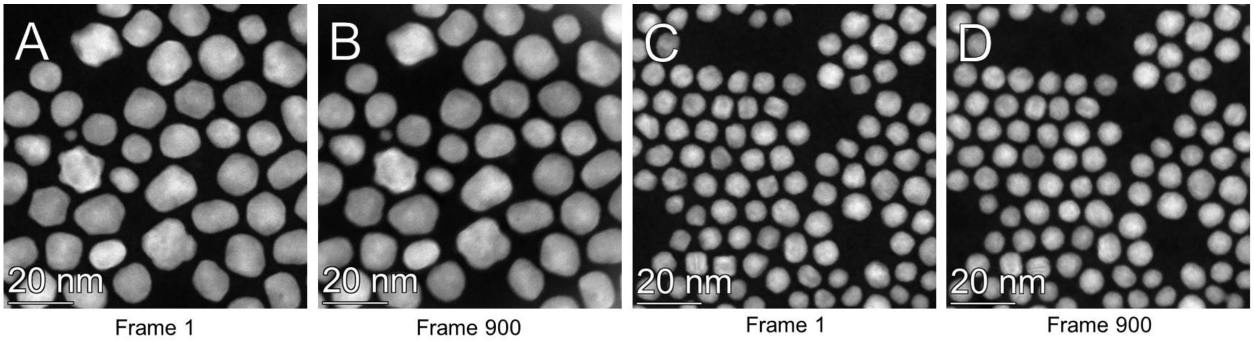

Figure S10. HAADF-STEM images corresponding to the first and final frame of EDX map acquisition for (A, B) CoNiCuPdPt Core@Shell NPs and (C,D) CoNiCuPdPt alloy NPs.

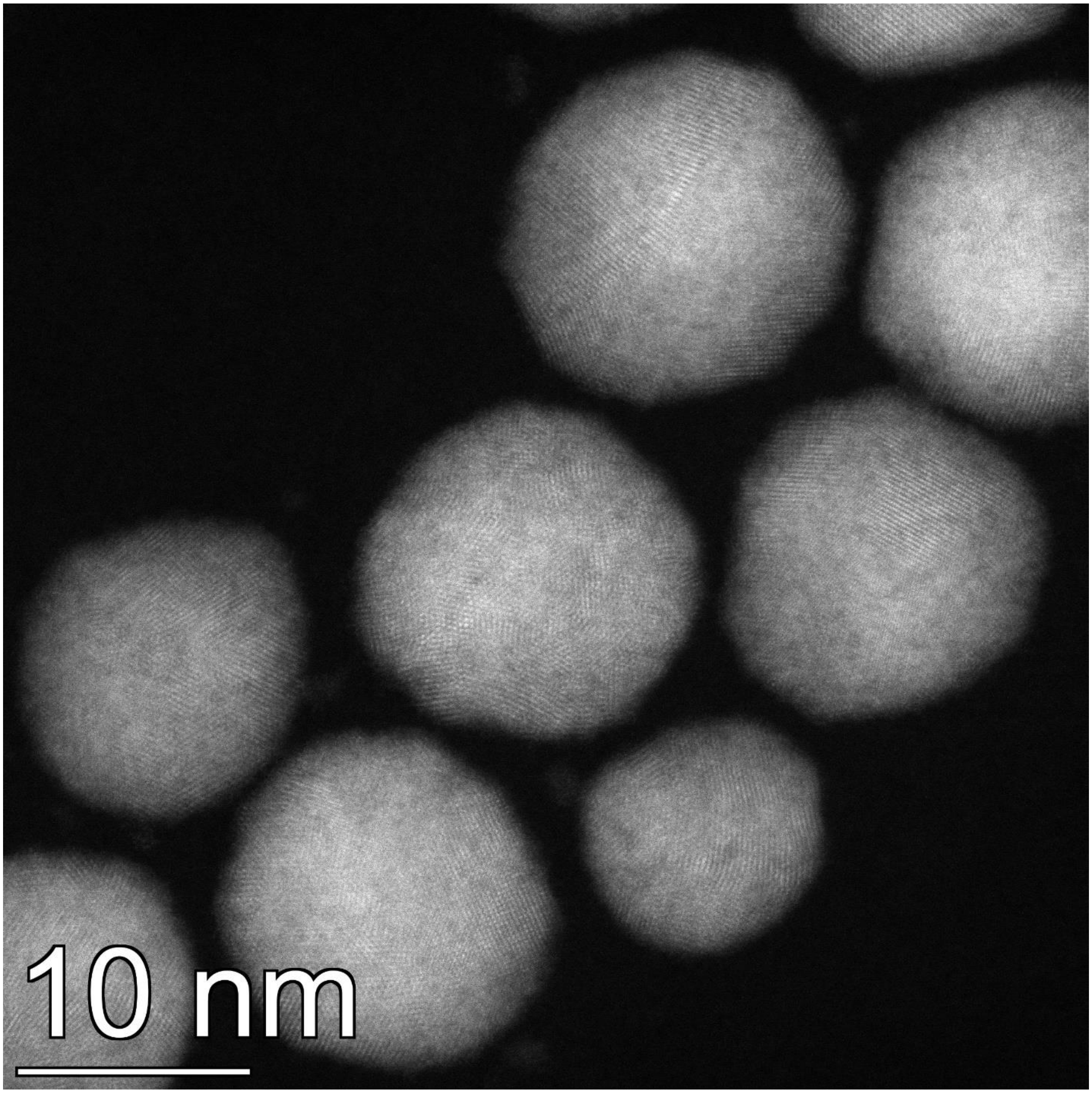

Figure S11. Additional HAADF-STEM image of the AuPd alloy NPs produced using the 0.2 nmol/sec.

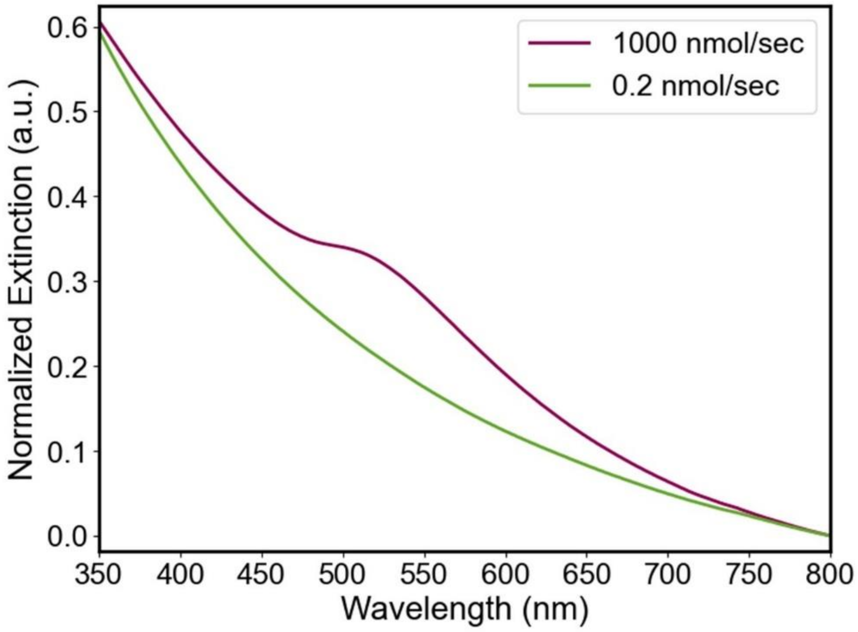

Figure S12. Extinction spectra of the Au@Pd NPs synthesized using the 1000 nmol/sec addition rate (red trace) and AuPd alloy NPs synthesized using the 0.2 nmol/sec addition rate (green trace).

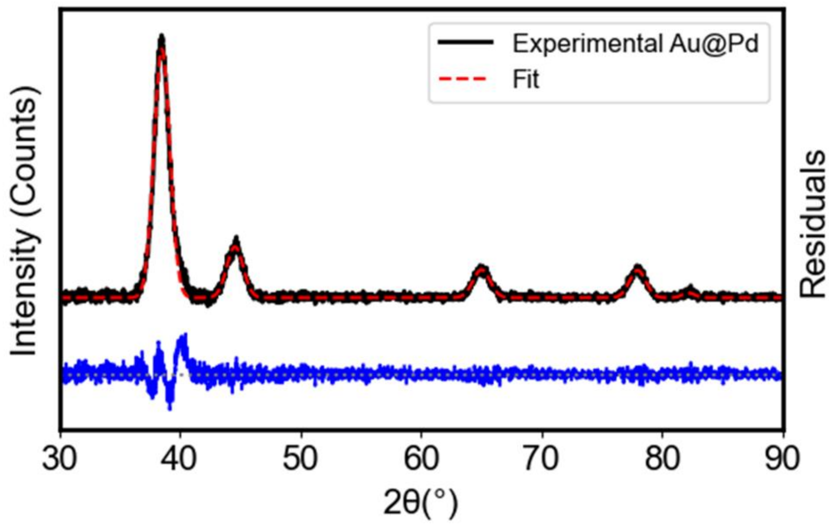

Figure S13. Experimental PXRD pattern of Au@Pd NPs (black trace) and corresponding fit (red trace) used to determine peak positions. The residuals from the fit are shown as the blue trace.

Table S3. Optimized peak positions and calculated lattice parameters of Au@Pd NPs from the PXRD pattern shown in Figure S13.

Optimized Position (2θ)	hkl	a (Å)
38.53983	111	4.04
44.60291	200	4.05
65.06215	220	4.05
78.00708	311	4.05
82.24274	222	4.05

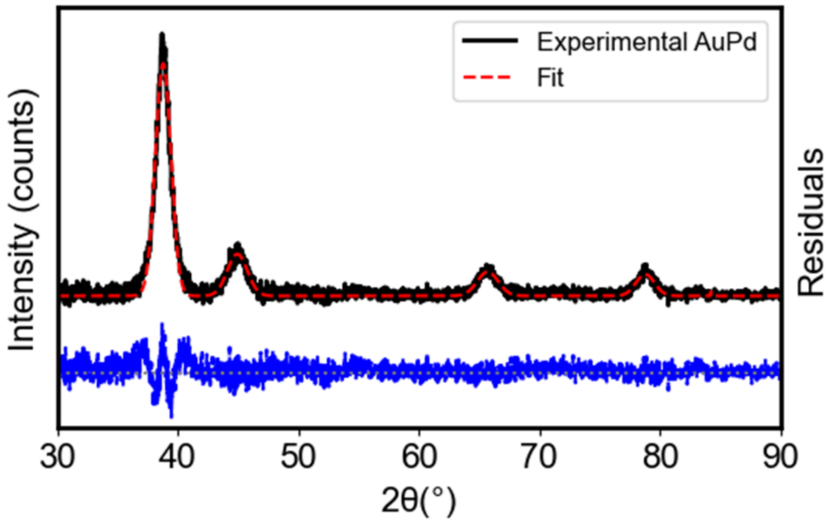

Figure S14. Experimental PXRD pattern of AuPd alloy NPs (black trace) and corresponding fit (red trace) used to determine the peak positions. The residuals from the fit are shown as the blue trace.

Table S4. Optimized peak positions and calculated lattice parameters of the AuPd alloy NPs from the PXRD pattern shown in Figure S14.

Optimized Position (2θ)	hkl	a (Å)
38.9078	111	4.00
45.0450	200	4.00
65.8790	220	4.00
79.3660	311	4.00
84.4786	222	3.98

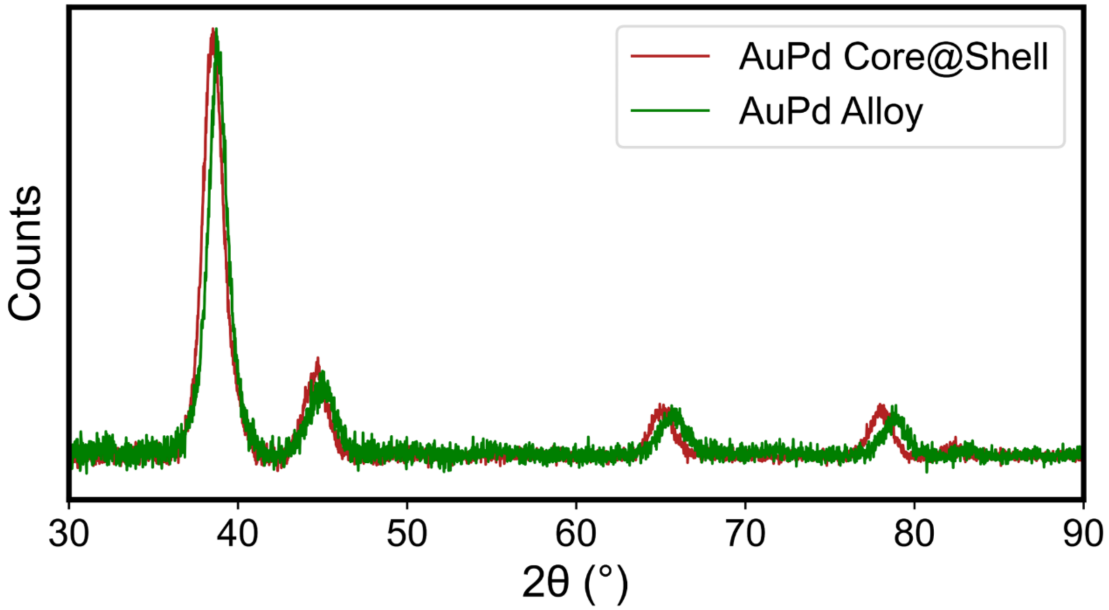

Figure S15. Overlay of PXRD patterns from Figures S13 and S14, where Au@Pd (red) and AuPd alloy NPs (green trace) are shown together. Due to the peak broadening induced by the crystallite size, visualizing the shift in the diffraction pattern is aided by this representation.

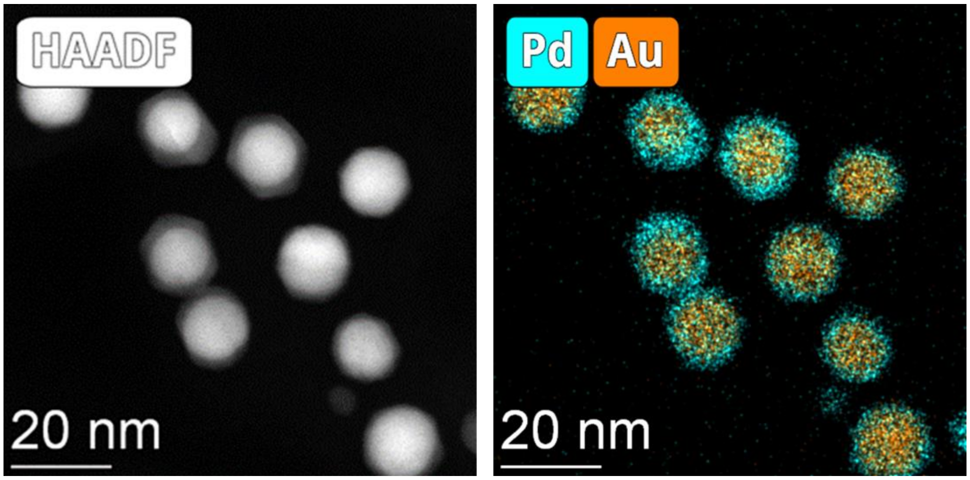

Figure S16. EDX images of Au@Pd NPs synthesized using an addition rate of 1000 nmol/sec followed by annealing at 220°C for six hours. Images indicate evolution in Pd shell faceting, but the core@shell structure is preserved in all cases and no evidence of alloying was observed.

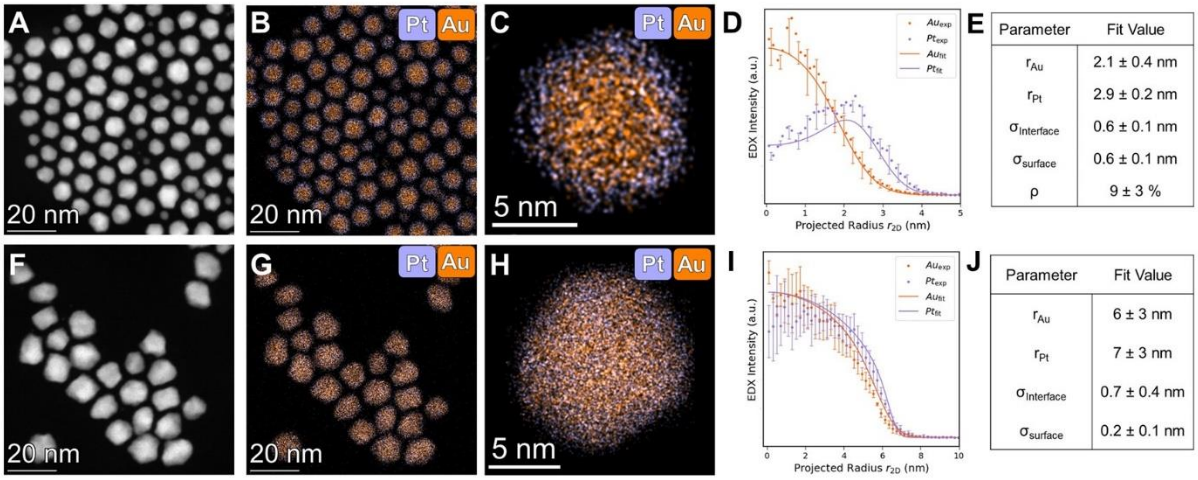

Figure S17. Structural characterization of bimetallic Au-Pt NPs produced using the rapid (1000 nmol/sec, A-E) and slow (0.2 nmol/sec, F-J) addition rates. (A) Low magnification HAADF-STEM image and (B) corresponding EDX map of Au@Pt NPs produced using a rapid precursor addition rate of 1000 nmol/sec. (C) EDX map of single Au@Pt core@shell NP (D) Experimental radial profile analysis of each element within the NP, along with the analytical fit to the data (solid lines). (E) Optimized parameters derived from the fit, supporting the core@shell assignment. (F) Low magnification HAADF-STEM image and (G) corresponding EDX map of AuPt alloy NPs produced using the slow addition rate of 0.2 nmol/sec. (H) EDX map of single AuPt alloy NP. (I) Experimental radial profile analysis of each element within the NP, along with the analytical fit to the data (solid lines). (J) Optimized parameters derived from the fit, supporting the alloy assignment. Platinum (Pt Lα) signals are displayed in purple and gold (Au Lα) signals in orange.

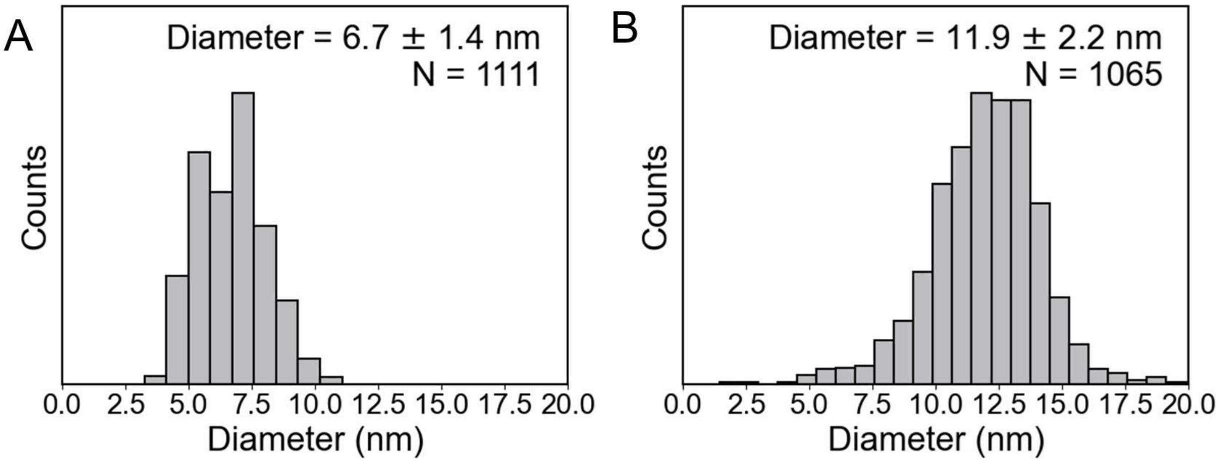

Figure S18. Size histograms for (A) Au@Pt NPs synthesized using the 1000 nmol/sec addition rate and (B) AuPt alloy NPs synthesized using the 0.2 nmol/sec addition rate.

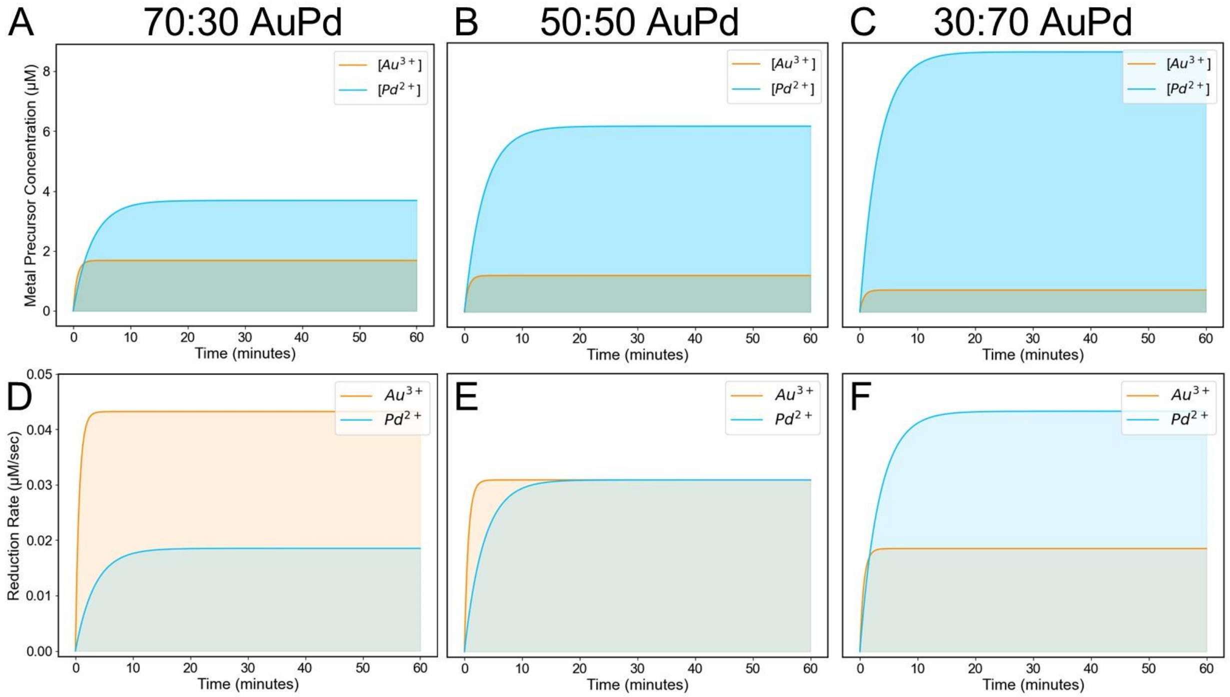

Figure S19. (A-C) Plots of the simulated instantaneous metal precursor concentrations and (D-F) corresponding reduction rates of Au^{3+} and Pd^{2+} when the metal precursors are added using the slow precursor addition rate (0.4 nmol/sec, total precursor). Here, we predict that changing the initial molar ratio of Au^{3+} to Pd^{2+} will impact the relative instantaneous ion concentrations, and therefore the instantaneous reduction rates accordingly, while still facilitating their synchronous reduction.

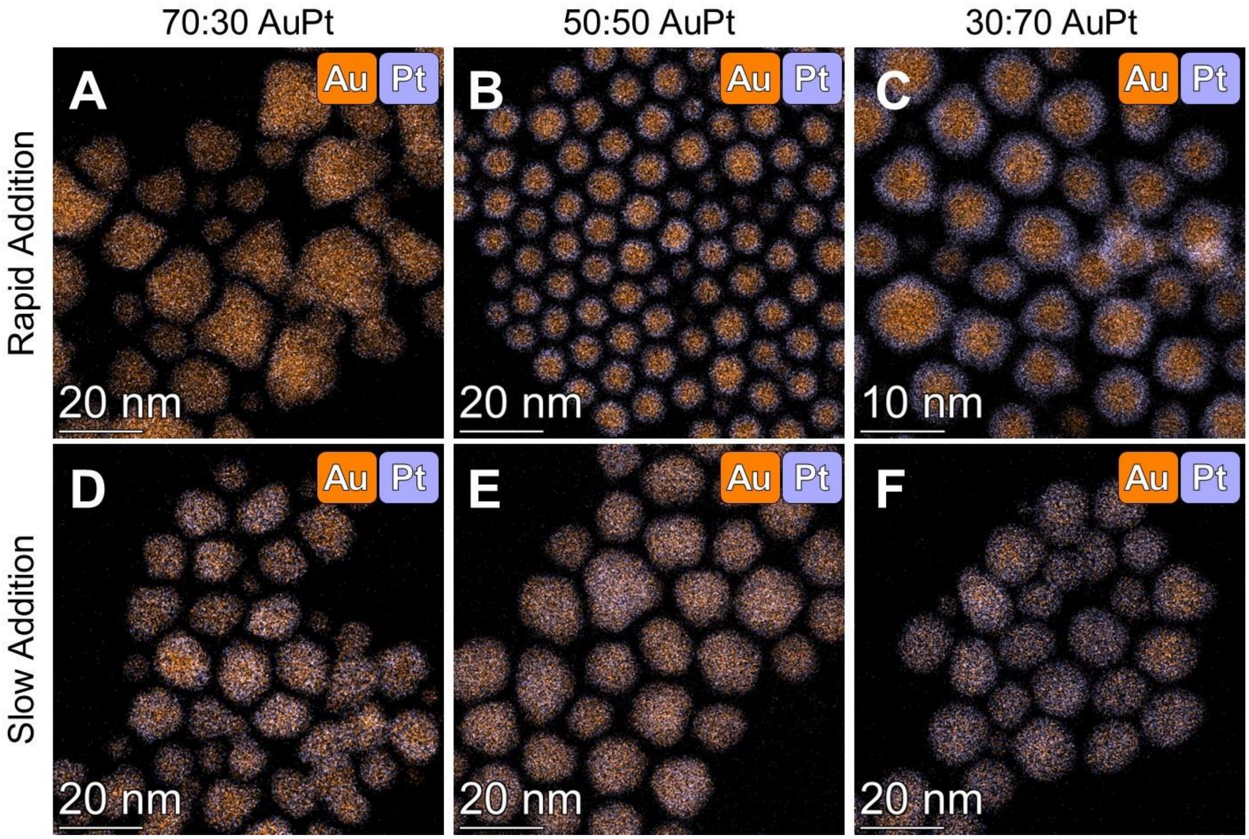

Figure S20. EDX maps of AuPt NPs produced with different stoichiometries of Au:Pt using a (A-C) rapid addition rate (2,000 nmol/sec, total precursor) and (D-F) slow addition rate (0.4 nmol/sec, total precursor). Platinum (Pt Lα) signals are displayed in purple and gold (Au Lα) signals in orange.

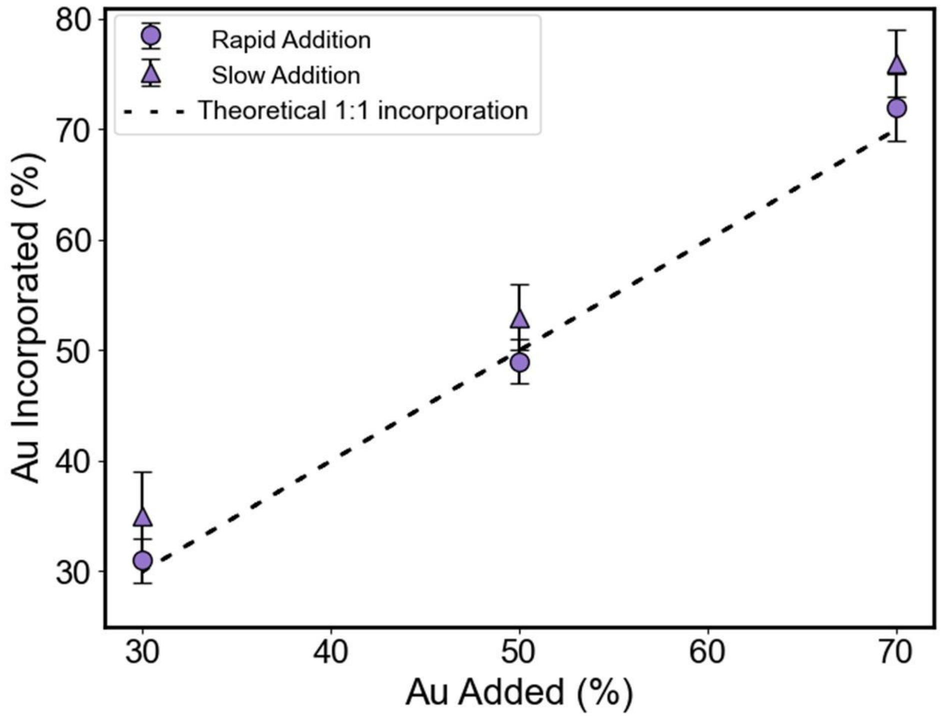

Figure S21. Percent Au incorporated into the Au-Pt NP samples as a function of the initial molar percent Au added to the synthesis for both the rapid (circles) and slow (triangles) addition rates. The dashed line represents the theoretical stoichiometry assuming 1:1 incorporation. As in the case of the Au-Pd particles, nominal and incorporated stoichiometries agree well for both the core@shell and alloyed NPs.

Table S5. Composition outcomes for Au-Pd NPs synthesized using either rapid (2,000 nmol/sec, total precursor) or slow (0.4 nmol/sec, total precursor) addition rates as determined by both single particle (EDX) and ensemble (ICP-OES) analyses.

Addition Rate	% Au Added	Composition (% Au) EDX (N ≥ 25)	Composition (% Au) ICP (N ≥ 3)
Rapid	30	30 ± 1	34 ± 4
	40	38 ± 3	38 ± 6
	50	54 ± 4	51 ± 2
	60	60 ± 2	61 ± 1
	70	73 ± 4	69 ± 5
Slow	30	34 ± 4	32 ± 5
	40	42 ± 2	43 ± 4
	50	50 ± 2	53 ± 2
	60	61 ± 4	62 ±3
	70	68 ± 3	71 ± 3

Table S6. Composition outcomes for Au-Pt NPs synthesized using either rapid (2,000 nmol/sec, total precursor) or slow (0.4 nmol/sec, total precursor) addition rates as determined by both single particle (EDX) and ensemble (ICP-OES) analyses.

Addition Rate	% Au Added	Composition (% Au) EDX (N ≥ 25)	Composition (% Au) ICP (N ≥ 3)
Rapid	30	36 ± 3	31 ± 2
	50	52 ± 4	49 ± 2
	70	69 ± 3	72 ± 3
Slow	30	35 ± 3	35 ± 4
	50	51 ± 3	53 ± 3
	70	71 ± 4	76 ± 3

Table S7. Physicochemical Properties of Metals in the Quinary System

Metal	Lattice Constant[11] (Å)	Atomic Radii[11] (Å)	Melting Point[11] (°C)	Redox Reaction	E°[12] (V vs SHE)
Co	3.55	2.00	1538	$Co^{2+} + 2e^{-} \rightarrow Co$	-0.28
Ni	3.52	1.97	1455	$Ni^{2+} + 2e^{-} \rightarrow Ni$	-0.26
Cu	3.61	1.96	1085	$Cu^{2+} + 2e^{-} \rightarrow Cu$	0.34
Pd	3.89	2.10	1554	$Pd^{2+} + 2e^{-} \rightarrow Pd$	0.95
Pt	3.92	2.13	1772	$Pt^{2+} + 2e^{-} \rightarrow Pt$	1.18

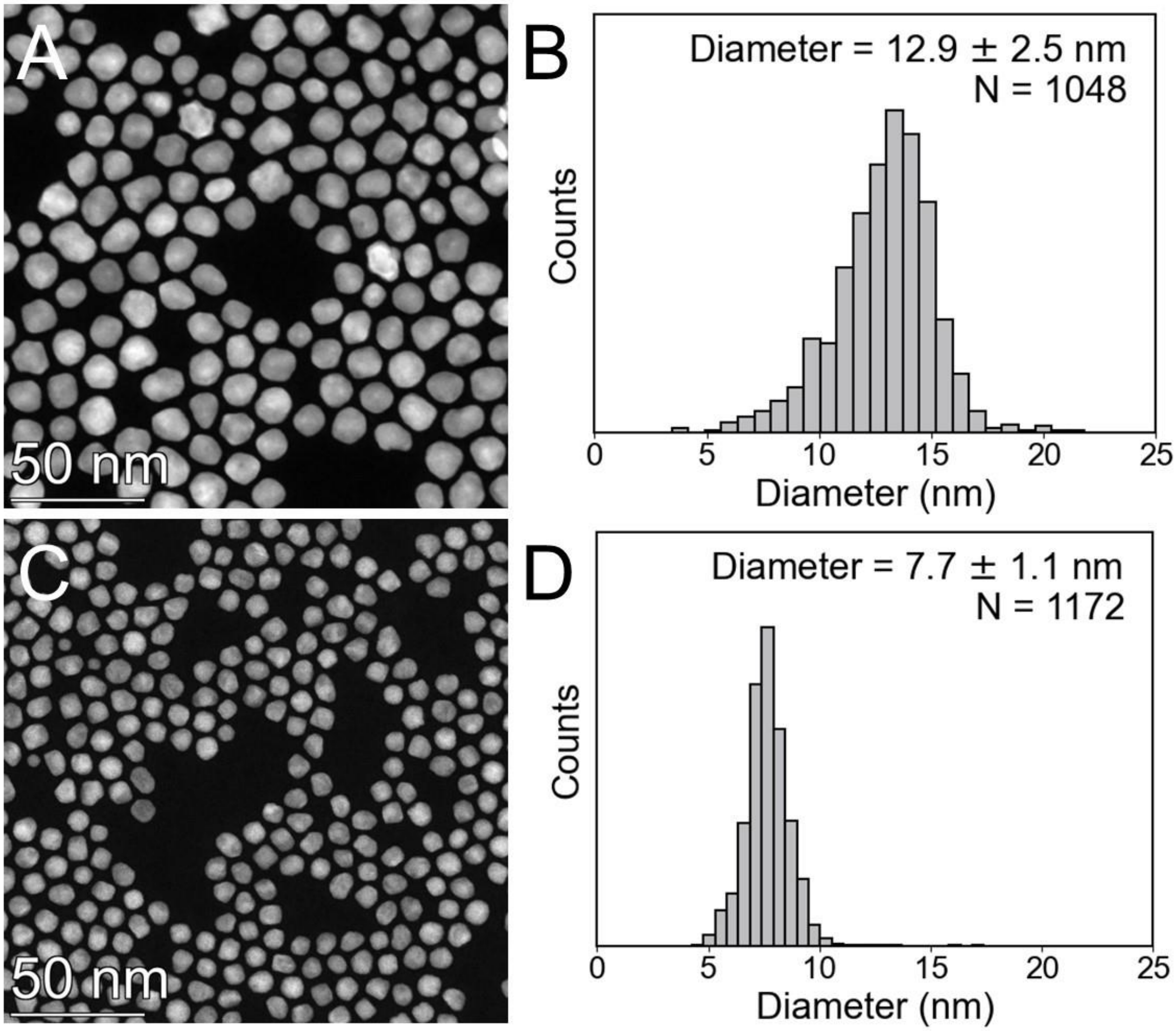

Figure S22. Low magnification STEM images and corresponding size histograms for (A, B) CoNiCuPdPt core@shell NPs synthesized using a rapid addition rate and (C, D) CoNiCuPdPt alloyed NPs synthesized using a slow addition rate.

Table S8. Composition of quinary NPs synthesized using both rapid and slow addition rates as determined by both single particle (EDX) and ensemble (ICP) analyses.

Addition Rate	Method	Co (%)	Ni (%)	Cu (%)	Pd (%)	Pt (%)
Rapid (10,000 nmol/sec)	ICP (N ≥ 3)	8 ± 4	14 ± 4	24 ± 2	28 ± 3	27 ± 2
	EDX (N ≥ 50)	8 ± 4	12 ± 4	25 ± 4	28 ± 4	27 ± 2
Slow (0.2 nmol/sec)	ICP (N ≥ 3)	12 ± 4	11 ± 3	21 ± 3	29 ± 3	27 ± 2
	EDX (N ≥ 50)	14 ± 9	8 ± 2	20 ± 7	32 ± 3	26 ± 2

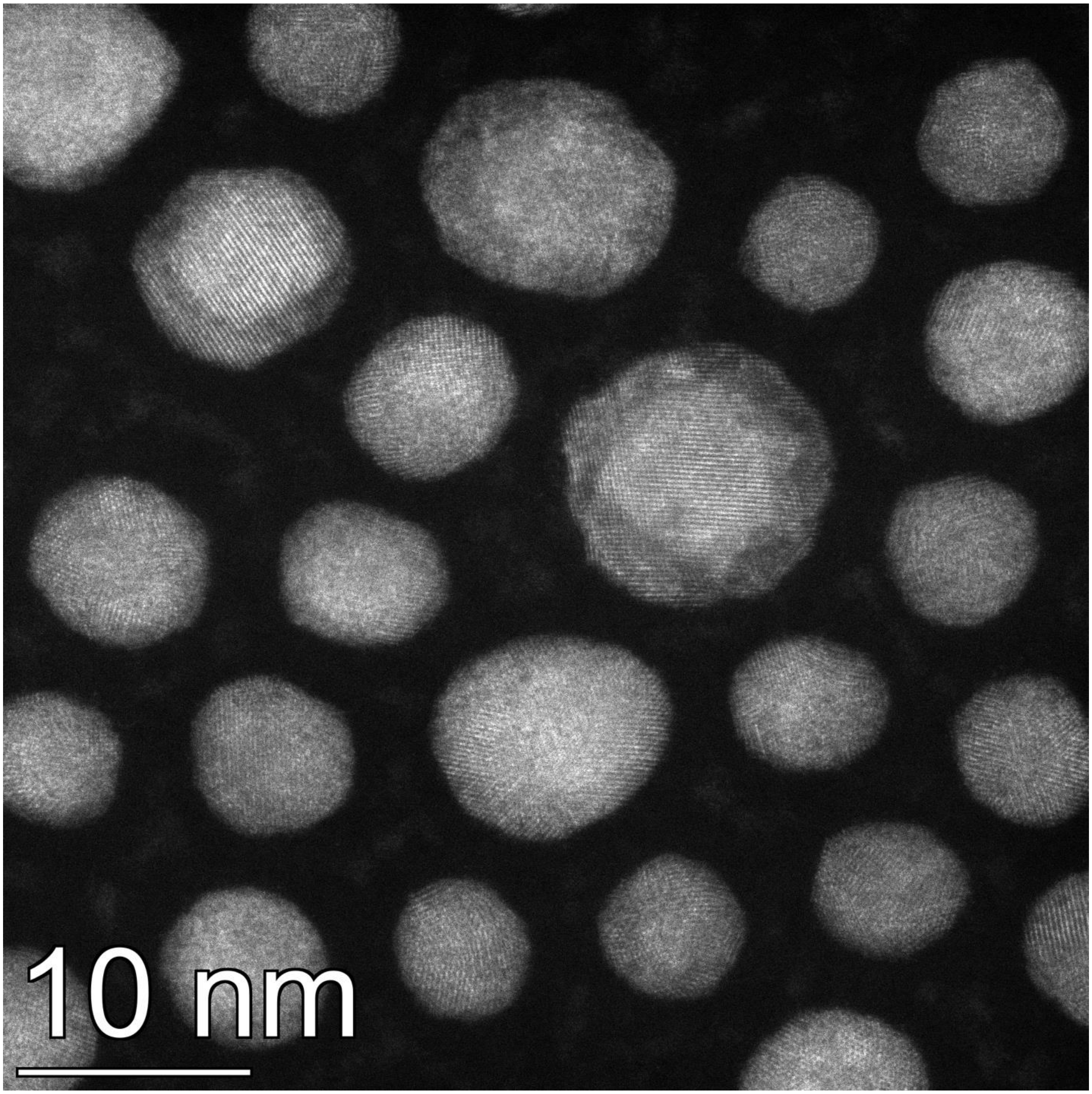

Figure S23. Additional HAADF-STEM image of the CoNiCuPdPt NPs produced using the rapid addition rate of 10,000 nmol/sec.

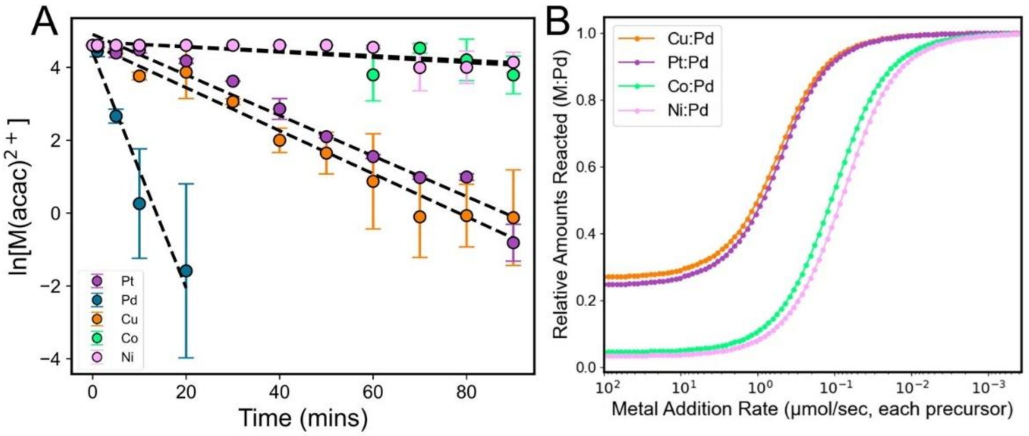

Figure S24. Analysis of the relative reduction kinetics in the synthesis of quinary CoNiCuPdPt NPs. Plots of ln[M^{2+}] (M = Co, Ni, Cu, Pd, or Pt) as a function of time with fits assuming pseudo first-order reaction kinetics (A). Here, the relative reduction kinetics we observe (Pd > Cu > Pt > Co, Ni) are consistent with the chemical arrangement observed in the core@shell structures produced using a rapid addition rate. (B) Plot of the predicted amounts of Co^{2+}, Ni^{2+}, Cu^{2+} or Pt^{2+} reacted relative to the amount of Pd^{2+} reacted during a given metal addition period as a function of precursor addition rate and used to predict the addition rate for alloy production.

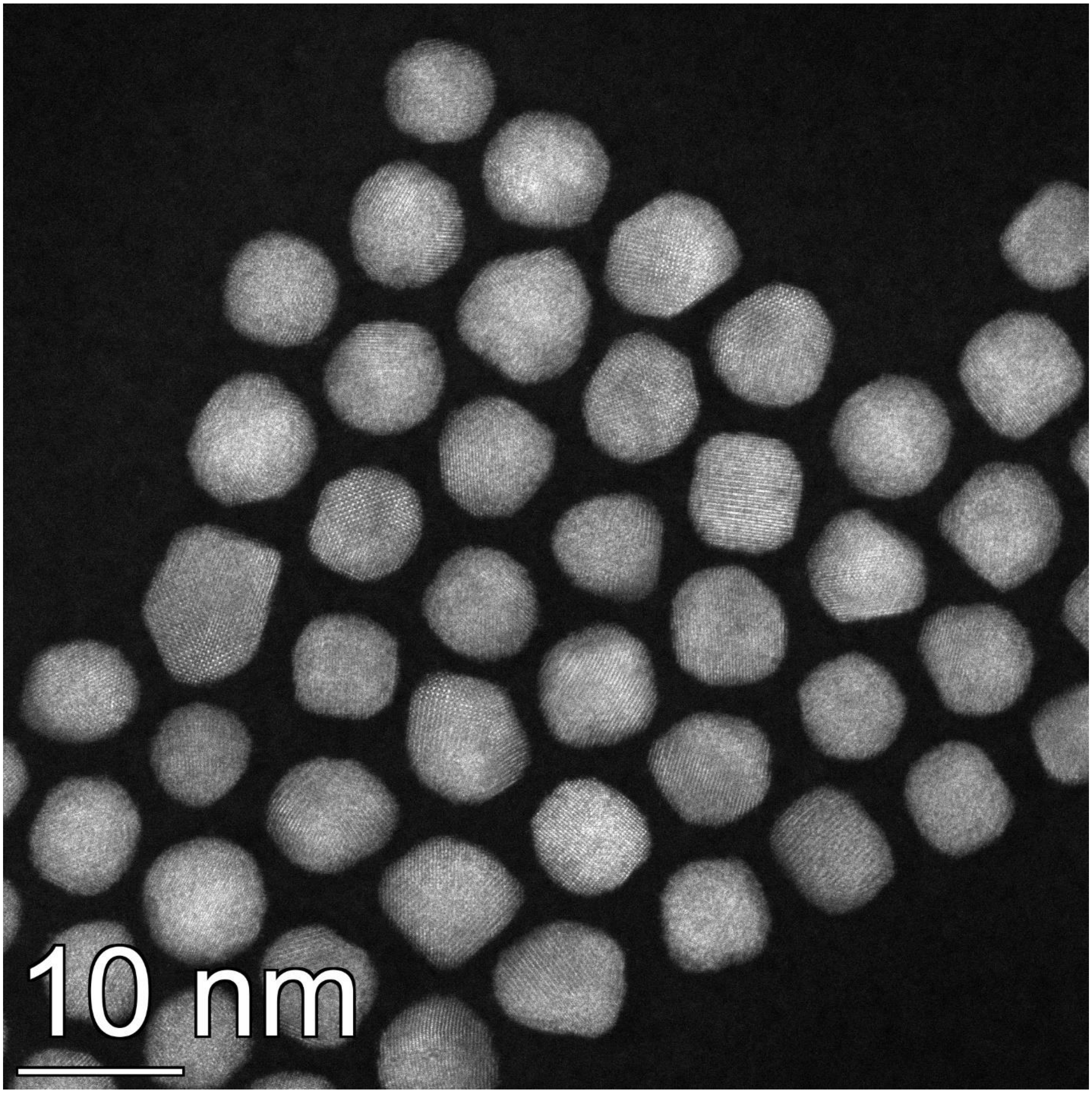

Figure S25. Additional HAADF-STEM image of the CoNiCuPdPt NPs produced using the slow addition rate of 2.27 nmol/sec, per precursor.

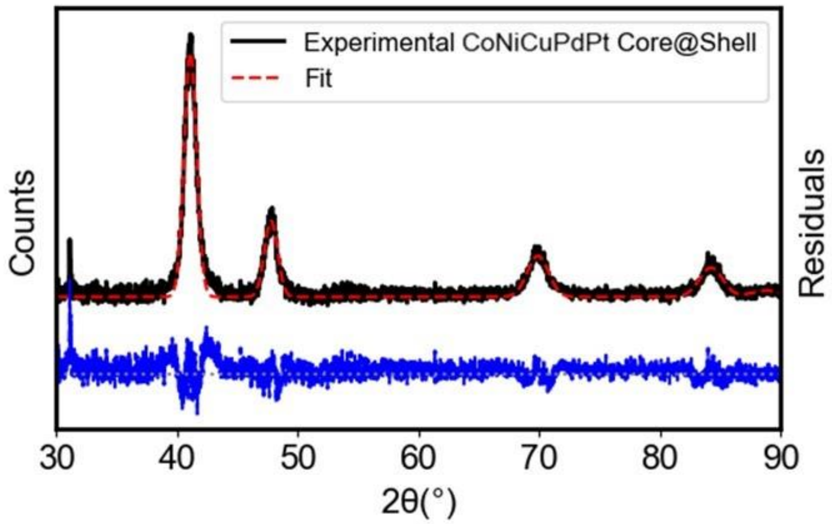

Figure S26. Experimental PXRD pattern of core@shell CoNiCuPdPt NPs synthesized using the rapid addition rate of 10,000 nmol/sec (black trace) and corresponding fit (red trace) used to determine the peak positions. The residuals from the fit are shown as the blue trace.

Table S9. Optimized peak positions and calculated lattice parameters from the PXRD fit of the core@shell CoNiCuPdPt NPs shown in Figure S26 synthesized using the rapid addition rate of 10,000 nmol/sec.

Optimized Position (2θ)	hkl	a (Å)
41.097	111	3.80
47.775	200	3.80
69.880	220	3.80
84.216	311	3.80
88.807	222	3.81

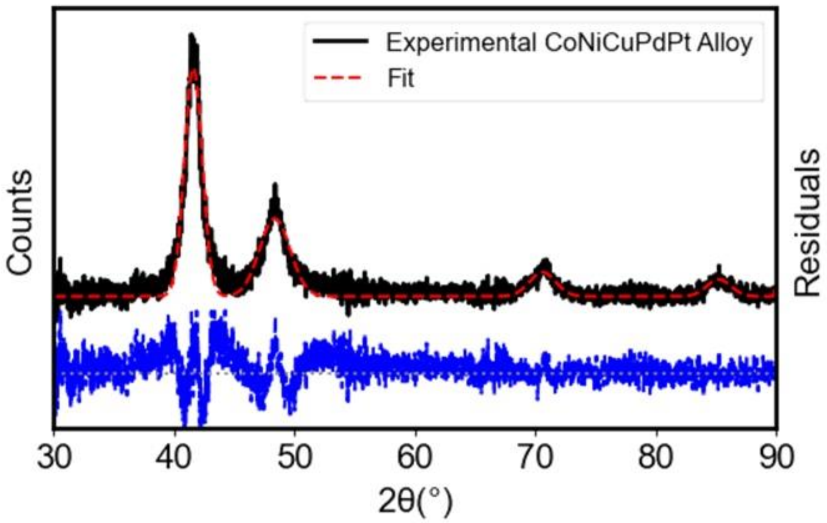

Figure S27. Experimental PXRD pattern of alloyed CoNiCuPdPt NPs synthesized using the slow addition rate of 2.27 nmol/sec (black trace) and corresponding fit (red trace) used to determine the peak positions. The residuals from the fit are shown as the blue trace.

Table S10. Optimized peak positions and calculated lattice parameters from the PXRD fit of pattern shown in Figure S27 from CoNiCuPdPt NPs synthesized using the slow addition rate of 2.27 nmol/sec.

Optimized Position (2θ)	hkl	a (Å)
41.585	111	3.76
48.349	200	3.76
70.580	220	3.76
85.212	311	3.77
89.804	222	3.78

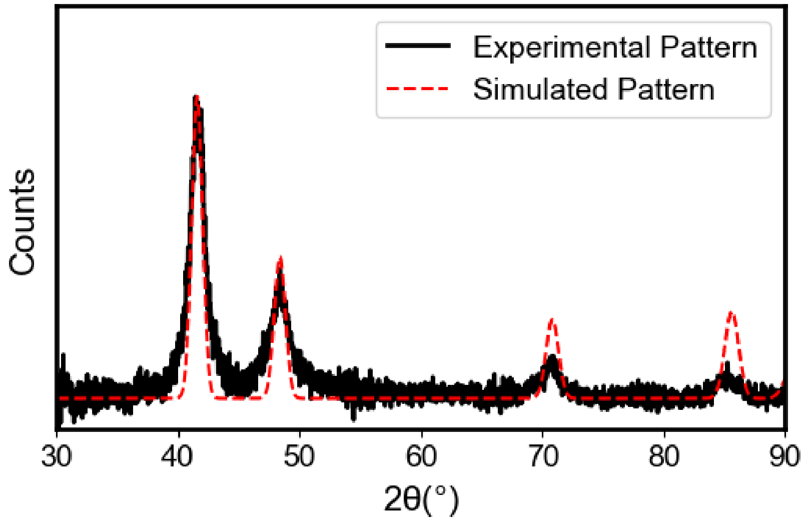

Figure S28. Experimental PXRD pattern of alloy CoNiCuPdPt NPs (black trace) compared to a simulated pattern assuming no off-site displacements (red trace). Simulated pattern was generated using Crystal Maker® version 11.0.2.

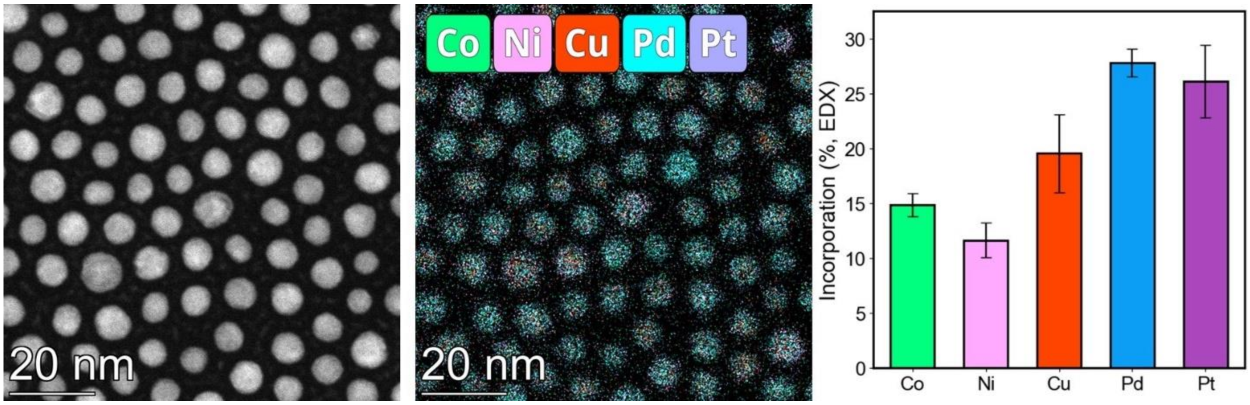

Figure S29. HAADF-STEM and corresponding EDX maps for CoNiCuPdPt core@shell NPs after annealing at the reaction temperature for 2 additional hours. Here, increasing the reaction time did not increase the incorporation of Co and Ni (incorporation determined by area EDX analysis).

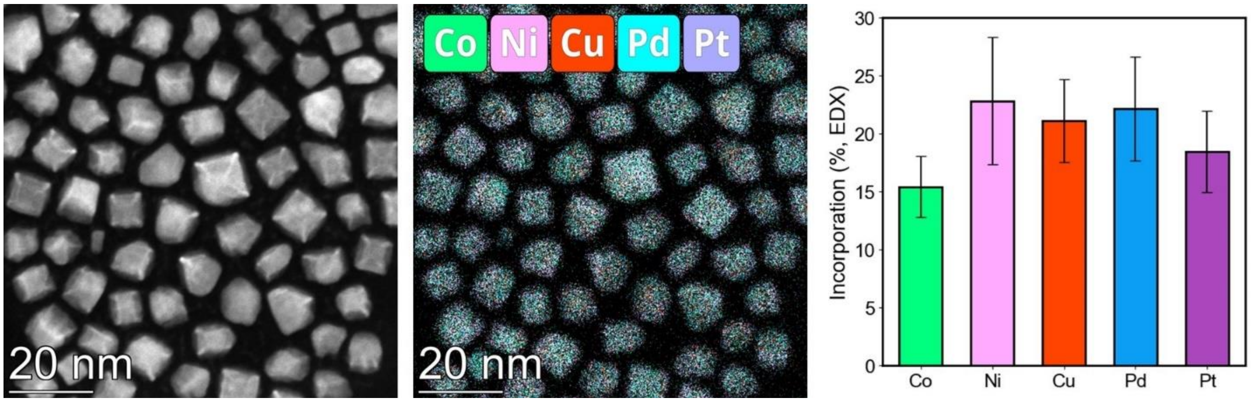

Figure S30. HAADF-STEM and corresponding EDX maps for CoNiCuPdPt alloyed NPs synthesized using more Co and Ni. Here, increasing the initial mol fraction of the Co and Ni precursors increased their incorporation, approaching equimolar distributions (incorporation determined by area EDX analysis). We note the change in particle morphology from pseudo-spherical to primarily faceted. The origins of this shape transition are a topic of ongoing investigation in our laboratory, however we note that the transition in NP topology is not correlated with a change in metal mixing behavior observed, the topic of our current study.

References

(1) Yang, T.-H.; Gilroy, K. D.; Xia, Y. Reduction Rate as a Quantitative Knob for Achieving Deterministic Synthesis of Colloidal Metal Nanocrystals. *Chem. Sci.* **2017**, *8* (10), 6730–6749. https://doi.org/10.1039/C7SC02833D.
(2) Yang, T.-H.; Peng, H.-C.; Zhou, S.; Lee, C.-T.; Bao, S.; Lee, Y.-H.; Wu, J.-M.; Xia, Y. Toward a Quantitative Understanding of the Reduction Pathways of a Salt Precursor in the Synthesis of Metal Nanocrystals. *Nano Lett.* **2017**, *17* (1), 334–340. https://doi.org/10.1021/acs.nanolett.6b04151.
(3) Held, J. T.; Hunter, K. I.; Dahod, N.; Greenberg, B.; Reifsnyder Hickey, D.; Tisdale, W. A.; Kortshagen, U.; Mkhoyan, K. A. Obtaining Structural Parameters from STEM–EDX Maps of Core/Shell Nanocrystals for Optoelectronics. *ACS Appl. Nano Mater.* **2018**, *1* (2), 989–996. https://doi.org/10.1021/acsanm.7b00398.
(4) Phillips, G. R.; Eyring, E. M. Error Estimation Using the Sequential Simplex Method in Nonlinear Least Squares Data Analysis. *Anal. Chem.* **1988**, *60* (8), 738–741. https://doi.org/10.1021/ac00159a002.
(5) Olsson, D. M.; Nelson, L. S. The Nelder-Mead Simplex Procedure for Function Minimization. *Technometrics* **1975**, *17* (1), 45–51. https://doi.org/10.1080/00401706.1975.10489269.
(6) Egerton, R. F. Dose Measurement in the TEM and STEM. *Ultramicroscopy* **2021**, *229*, 113363. https://doi.org/10.1016/j.ultramic.2021.113363.
(7) Chen, S.; Zhang, X.; Zhao, J.; Zhang, Y.; Kong, G.; Li, Q.; Li, N.; Yu, Y.; Xu, N.; Zhang, J.; Liu, K.; Zhao, Q.; Cao, J.; Feng, J.; Li, X.; Qi, J.; Yu, D.; Li, J.; Gao, P. Atomic Scale Insights into Structure Instability and Decomposition Pathway of Methylammonium Lead

Iodide Perovskite. *Nat. Commun.* **2018**, *9* (1), 4807. https://doi.org/10.1038/s41467-018-07177-y.
(8) Johnston-Peck, A. C.; DuChene, J. S.; Roberts, A. D.; Wei, W. D.; Herzing, A. A. Dose-Rate-Dependent Damage of Cerium Dioxide in the Scanning Transmission Electron Microscope. *Ultramicroscopy* **2016**, *170*, 1–9. https://doi.org/10.1016/j.ultramic.2016.07.002.
(9) Egerton, R. F.; Li, P.; Malac, M. Radiation Damage in the TEM and SEM. *Micron* **2004**, *35* (6), 399–409. https://doi.org/10.1016/j.micron.2004.02.003.
(10) Egerton, R. F.; McLeod, R.; Wang, F.; Malac, M. Basic Questions Related to Electron-Induced Sputtering in the TEM. *Ultramicroscopy* **2010**, *110* (8), 991–997. https://doi.org/10.1016/j.ultramic.2009.11.003.
(11) *CRC Handbook of Chemistry and Physics: A Ready-Reference Book of Chemical and Physical Data*, 92nd ed., 2011–2012.; Haynes, W. M., Lide, D. R., Eds.; CRC Press: Boca Raton, Fla., 2011.
(12) Bard, A. J.; Parsons, R.; Jordan, J. *Standard Potential in Aqueous Solutions*; Monographs in electroanalytical chemistry and electrochemistry; M. Dekker: New York Basel, 1985.
